# Supplementary material for: A regulatory variant impacting TBX1 expression contributes to basicranial morphology in Homo sapiens
Source: Am J Hum Genet. 2024 Apr 11;111(5):939–53. doi: 10.1016/j.ajhg.2024.03.012 (PMC11080286; doi:10.1016/j.ajhg.2024.03.012)
Supplement: Document S1. Figures S1–S10 and supplemental methods [file mmc1.pdf]

The American Journal of Human Genetics, Volume *111*

Supplemental information

A regulatory variant impacting *TBX1* expression contributes to basicranial morphology in *Homo sapiens*

Noriko Funato, Arja Heliövaara, and Cedric Boeckx

A

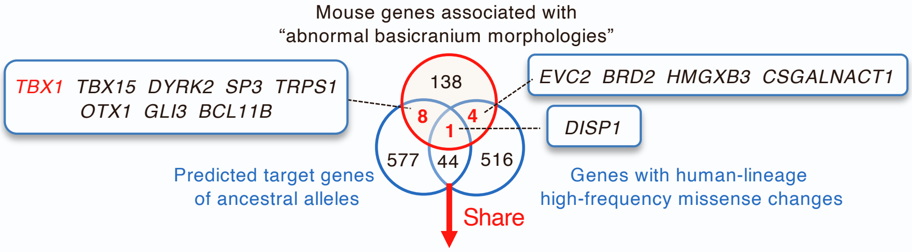

Abnormal basicranium morphologies of gene-engineered mice

Gene	Abnormal basicranium angle	Abnormal foramen magnum morphology	Abnormal basioccipital bone	Abnormal SOS	Abnormal basisphenoid bone	Abnormal ISS	Abnormal presphenoid bone	Short basicranium	References<br>PubMed ID<br>MGI Ref. ID
Evc2	N	N	N	N	+	+	+	+	23026747
Tbx1	+ (this study)	+ (this study)	+	+	+	N	N	+	32442036
Tbx15	N	+	+	N	N	N	N	NA	15652702
Dyrk2	N	N	+	+	+	+	+	+	32758357
Sp3	N	N	N	N	+	N	+	NA	11472836
Trps1	N	N	N	N	+	N	+	NA	31130868
Otx1	N	N	N	N	+	N	+	NA	9077465
Disp1	N	N	N	N	+	N	+	NA	15269168
Gli3	N	N	N	N	+	N	N	NA	11978771
Brd2	N	N	N	N	+	N	N	NA	MGI J:239583
Hmgxb3	N	N	N	N	+	N	N	NA	MGI J:239583
Csgalnact1	N	N	N	+	N	N	N	+	30459452
Bcl11b	N	N	N	N	N	N	N	+	23727454

Posterior ← Skull base → Anterior

B

SNP	Ref Allele	Alt Allele	Global	African	American	Europe	East Asian	South Asian
			Allele Frequencies (1000 Genomes Project)					
rs41300444	A	G	0.0160	0	0.003	0.0139	0.0218	0.043
rs41297812	C	T	0.0176	0.0030	0.003	0.0139	0.0258	0.043
rs72646954	T	C	0.0192	0.0091	0.004	0.0139	0.0258	0.042
rs80179718	A	G	0.0192	0.0091	0.004	0.0139	0.0258	0.042
rs41298798	C	G	0.0166	0	0.003	0.0139	0.0258	0.042
rs8137465	T	C	0.0162	0.0053	0.006	0.0129	0.0169	0.041
rs41300472	T	C	0.0160	0	0.003	0.0149	0.0218	0.042

C

rs41298798 (C/G)

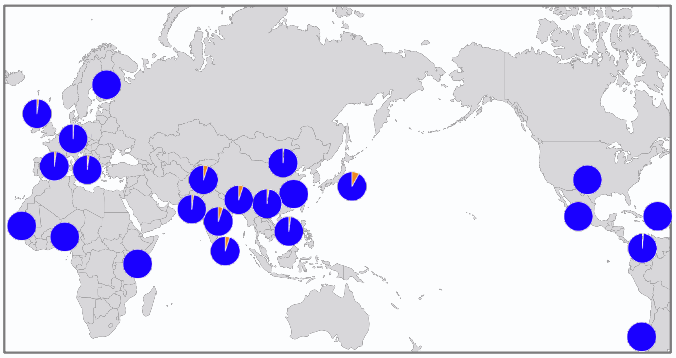

Figure S1. Scheme showing the strategy used to select candidate SNPs.
(A) Strategy for screening candidate genes that induce contemporary-human-specific morphology at the skull base. We used a list of predicted target genes of ancestral alleles,[1] a list of genes with human-lineage high-frequency missense changes,[2] and a list of mouse genes associated with "abnormal basicranium morphologies" obtained from the Mouse Genome Informatics (MGI) database. SOS, spheno-occipital synchondrosis; ISS, intersphenoid synchondrosis; N, not reported; NA, not available.
(B) SNPs at the *TBX1* locus. Ref, reference allele; Alt, alternative allele.
(C) Frequency distribution of rs41298798 in different populations superimposed on a world map. Blue represents the frequency of the C allele. The ancestral G allele (orange) is most common in Japan. Source data are shown in Table S2.

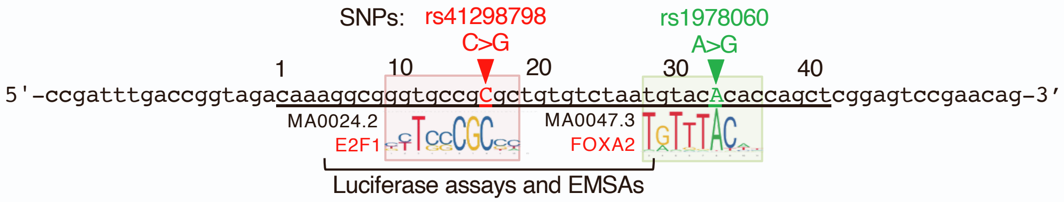

Figure S2. The sequence of a 41-bp deletion at the rs41298798 regulatory region includes rs1978060. Sequence of a 41-bp deletion at rs41298798, with two single-guide RNAs flanking the rs41298798 regulatory region. The variant rs1978060, which confers a significant genetic predisposition to adolescent idiopathic scoliosis in an East Asian population,[3] is located in a 17-bp 3′ region of rs41298798. EMSAs, Electrophoretic mobility shift assays.

rs41298798 rs1978060

1 10 20 30 40

5'-caaaggcgggtgccgCgctgtgtctaatgtacAcaccagct-3'

Matrix ID	Name	Score	Relative score	Start	End	Strand	Predicted sequence	
MA0767.1	GCM2	7.807088	0.850760689	3	12	+	aaggcgggtg	
MA0745.1	SNAI2	5.510656	0.856435377	5	13	+	ggcgggtgc	
MA0024.2	E2F1	5.776952	0.854934316	3	13	+	aaggcgggtgc	
MA0522.3	TCF3	9.523167	0.898157425	4	14	-	ggcacccgcct	
MA1648.1	TCF12	9.294592	0.888993111	4	14	-	ggcacccgcct	
MA0103.3	ZEB1	7.4171805	0.873963857	4	14	-	ggcacccgcct	
MA1099.2	HES1	5.41556	0.856389072	5	14	+	ggcgggtgcc	
MA0830.2	TCF4	10.570523	0.885564656	3	15	-	cggcacccgcctt	
MA1631.1	ASCL1	10.24938	0.880972334	3	15	-	cggcacccgcctt	
MA1583.1	ZFP57	10.4456	0.909558656	7	19	+	cgggtgccgCgct	rs41298798
MA0024.2	E2F1	5.5903487	0.852126923	9	19	-	agcGcggcacc	
MA1563.1	SOX18	6.839807	0.907395756	15	22	-	cacagcGc	
MA1647.1	PRDM4	9.553936	0.88333676	16	26	+	Cgctgtgtcta	
MA0442.1	SOX10	7.0969906	0.91952059	18	23	+	ctgtgt	
MA0498.2	MEIS1	6.205561	0.912160739	20	26	-	tagacac	
MA0033.1	FOXL1	5.5664096	0.857303304	19	26	-	tagacaca	
MA0723.2	VAX2	5.5537796	0.892951318	24	31	-	tacattag	
MA0032.1	FOXC1	5.460103	0.887481988	24	31	+	ctaatgta	
MA0875.1	BARX1	5.9942513	0.885961173	24	31	-	tacattag	
MA0876.1	BSX	5.3524723	0.876570725	24	31	-	tacattag	
MA1504.1	HOXC4	4.738065	0.876302534	24	31	-	tacattag	
MA0132.2	PDX1	5.527857	0.875079817	24	31	-	tacattag	
MA0723.2	VAX2	4.216518	0.863018615	24	31	+	ctaatgta	
MA1498.2	HOXA7	5.4942107	0.861343583	24	31	-	tacattag	
MA0710.1	NOTO	8.469622	0.91750614	23	32	-	gtacattaga	
MA0903.1	HOXB3	6.921685	0.895003802	23	32	-	gtacattaga	
MA0902.1	HOXB2	6.6082387	0.882637117	23	32	-	gtacattaga	
MA0900.1	HOXA2	5.6248317	0.857601958	23	32	-	gtacattaga	
MA0035.4	GATA1	7.874824	0.852245545	22	32	+	gtctaatgtac	
MA0888.1	EVX2	5.4408298	0.851964332	23	32	-	gtacattaga	
MA0849.1	FOXO6	8.234046	0.914004601	29	35	+	gtacAca	rs1978060
MA0157.1	FOXO3	9.379627	0.907205504	28	35	+	tgtacAca	
MA0848.1	FOXO4	7.686152	0.90060836	29	35	+	gtacAca	
MA0847.1	FOXD2	6.9104347	0.899571311	29	35	+	gtacAca	
MA0033.2	FOXL1	5.8368673	0.882262815	29	35	+	gtacAca	
MA0042.2	FOXI1	6.2587543	0.866962617	29	35	+	gtacAca	
MA0148.2	FOXA1	8.519514	0.86534325	25	35	-	tgTgtacatta	
MA0148.1	FOXA1	8.522632	0.864248817	25	35	-	tgTgtacatta	
MA0850.1	FOXP3	5.5353866	0.854954653	29	35	+	gtacAca	
MA0847.2	FOXD2	10.602455	0.900613175	24	36	+	ctaatgtacAcac	
MA0032.1	FOXC1	6.571134	0.949630275	30	37	-	ggtgTgta	
MA0047.3	FOXA2	10.523581	0.906065173	27	37	+	atgtacAcacc	
MA1103.2	FOXK2	10.31445	0.901509117	27	37	+	atgtacAcacc	
MA1683.1	FOXA3	10.009295	0.896010813	27	37	+	atgtacAcacc	
MA1103.1	FOXK2	9.9036875	0.892487828	27	37	+	atgtacAcacc	
MA0481.3	FOXP1	9.700128	0.892378259	27	37	+	atgtacAcacc	
MA0481.2	FOXP1	8.9081	0.860491841	27	38	+	atgtacAcacca	
MA0807.1	TBX5	7.183253	0.853436044	31	38	-	tggtgTgt	
MA0148.3	FOXA1	10.862134	0.912633721	25	39	-	ctggtgTgtacatta	
MA1563.1	SOX18	5.235231	0.86676277	32	39	+	cAcaccag	
MA1566.1	TBX3	7.0365567	0.865503127	30	39	-	ctggtgTgta	
MA0745.1	SNAI2	7.6557755	0.899224965	33	41	-	agctggtgT	
MA1558.1	SNAI1	8.469676	0.898710002	32	41	-	agctggtgTg	
MA0830.1	TCF4	6.542236	0.887485653	32	41	+	cAcaccagct	
MA0522.2	TCF3	6.4015374	0.877109288	32	41	+	cAcaccagct	

Figure S3. The predicted effect of the 41-bp deletion on transcription factor (TF) binding.
A total of 55 putative sites were found with a relative profile score threshold of 85% for all human TF motif sets using the JASPAR 2020 CORE data set.

SNP ID	Variant ID	Gene Symbol	*P*-value	Tissue
rs1978060	chr22_19762002_A_G_b38	*TBX1*	3.00E-18	Prostate
rs1978060	chr22_19762002_A_G_b38	*TBX1*	5.90E-07	Esophagus - Mucosa
rs1978060	chr22_19762002_A_G_b38	*GNB1L*	0.0000076	Brain - Nucleus accumbens (basal ganglia)
rs1978060	chr22_19762002_A_G_b38	*GNB1L*	0.00011	Thyroid
rs1978060	chr22_19762002_A_G_b38	*RTL10 / C22orf29*	0.000016	Brain - Caudate (basal ganglia)
rs1978060	chr22_19762002_A_G_b38	*RTL10 / C22orf29*	1.10E-18	Nerve - Tibial
rs1978060	chr22_19762002_A_G_b38	*AC000089.3*	0.0000036	Nerve - Tibial

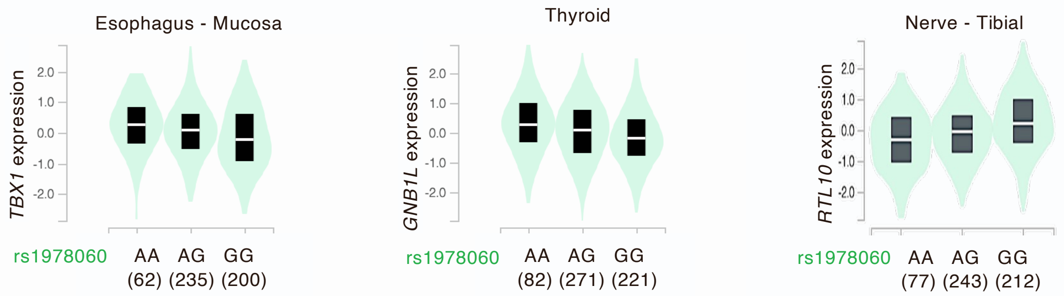

Figure S4. Expression quantitative trait locus (eQTL) analysis of all tissues shows that rs1978060 is associated with the expression levels of *TBX1*, *GNB1L*, *RTL10*, and *AC000089.3*.
GTEx was accessed on 29 June 2022.

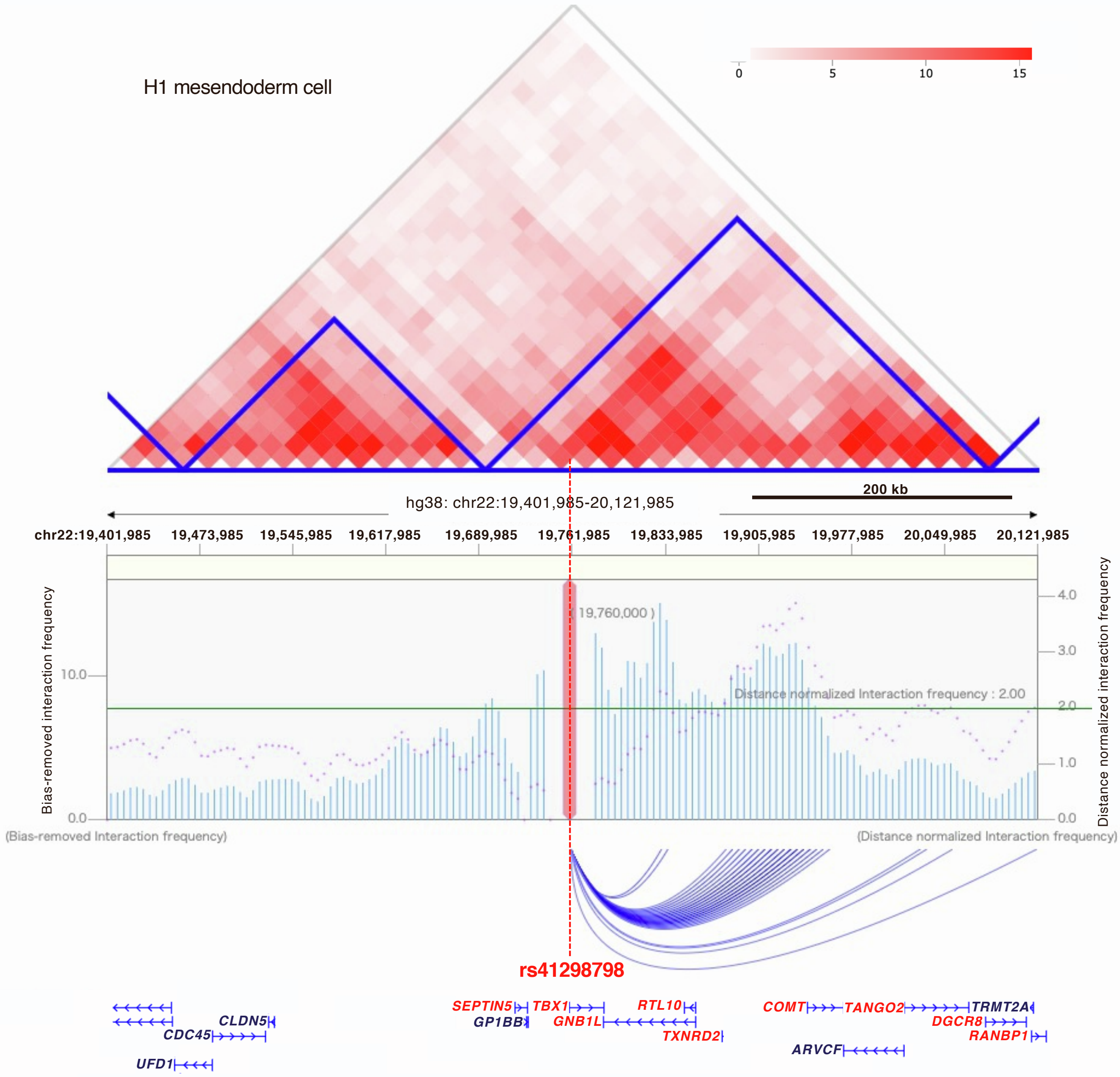

Figure S5. Contact between rs41298798 and genes at 22q11.21 based on the three-dimensional chromatin structure. Snapshot of the 3D Interaction Viewer (3DIV) with the hg38 genome assembly showing the genomic context of chromosome 22q11.21.

(Top) 20 kb resolution Hi-C data in H1 mesendoderm cells.

(Bottom) Coding RNAs. Strong interactions were observed between the region containing rs41298798 and promoters in the same topologically associating domain (blue triangle box). Genes shown in red have been analysed in Figure 2.

A

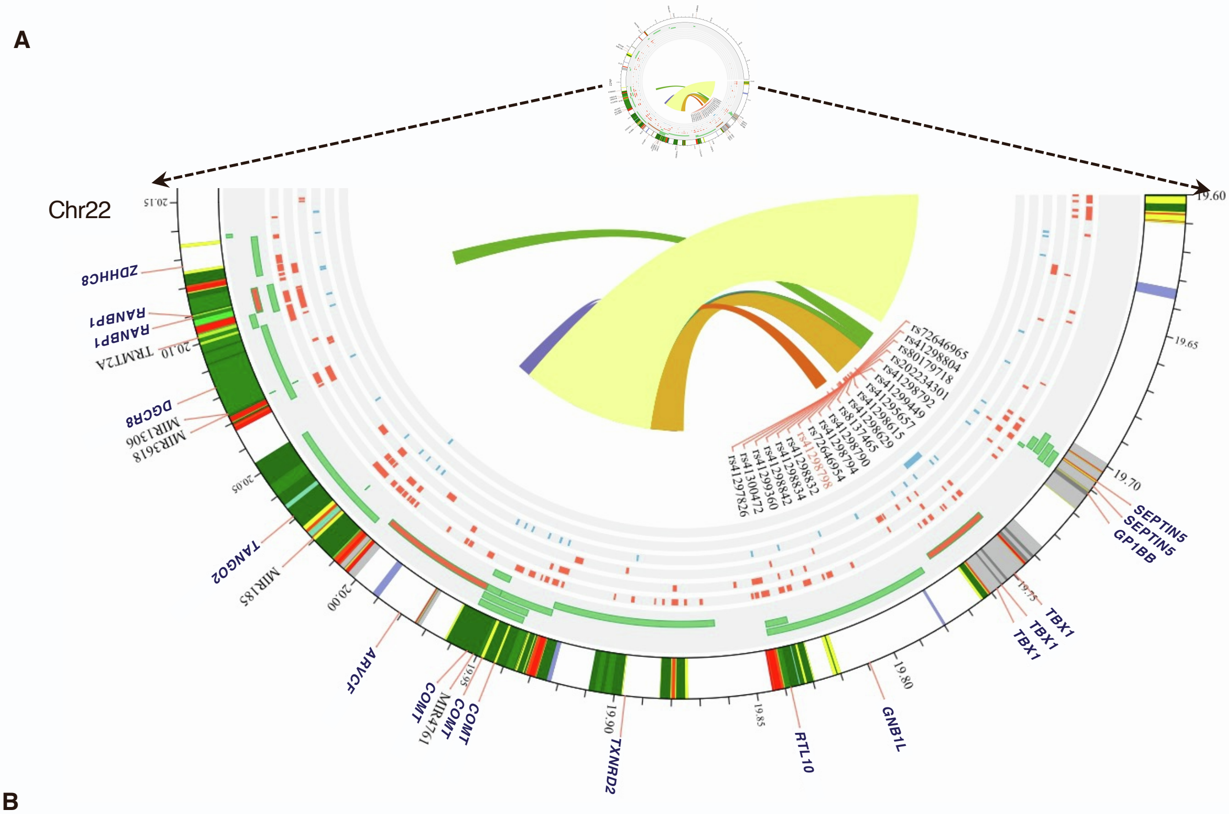

B

Gene	Loop type	Cell line	Tissue
SEPTIN5	Within loop	LNCAP	
SEPT5-GP1BB	Within loop	LNCAP	
GP1BB	Within loop	LNCAP GM12878 HepG2	Blood, Liver
TBX1	Within loop	SKMEL5 LNCAP THP1 HAP1 HepG2 KBM7 NHEK PANC1 GM12878 HCT116 IMR90 HMEC Caki2 H1-NPC HUVEC	Liver, Skin, Blood, Lung, Breast, Spleen, Pancreas, Blood vessel
GNB1L	Within loop	SKMEL5 THP1 HAP1 HepG2 T47D RPMI7951 NHEK PANC1 GM12878 HCT116 IMR90 HMEC Caki2 H1-NPC KBM7 A549 HUVEC	Liver, Psoas, Skin, Blood, Aorta, Spleen, Pancreas, Lung, Breast, Epithelium, Blood vessel
RTL10	Within loop	SKMEL5 THP1 HAP1 HepG2 T47D RPMI7951 NHEK PANC1 GM12878 LNCAP HCT116 IMR90 HMEC Caki2 H1-NPC KBM7 A549 HUVEC	Psoas, Lung, Liver, Skin, Aorta, Spleen, Pancreas, Blood, Breast, Epithelium, Blood vessel
TXNRD2	Within loop	LNCAP	
COMT	Within loop	LNCAP	

C

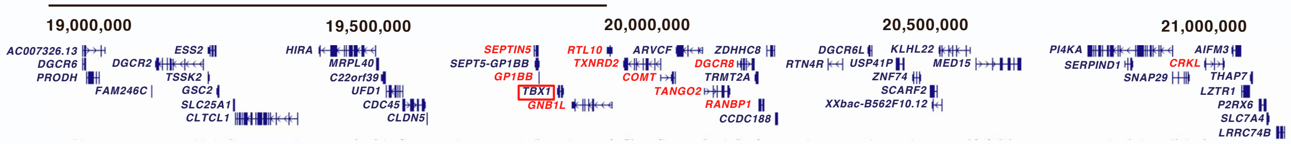

Figure S6. Long-range chromatin looping formed between the enhancer region containing the candidate causal SNP rs41298798.

(A) A circular plot of chromosome interactions related to rs41298798. In the circular plot, from outer to inner, the circle represents chromatin states, annotated genes, histone modification set (red; H3K4me1, H3K4me3, H3K27ac), TF set (blue; CTCF, CEBPB, CEBPD), current SNP (red) and associated SNPs, and three-dimensional (3D) chromatin interactions.[4]

(B) Genes that interact with rs41298798 through 3D chromatin loops in different cell types and tissues. Data were taken from 3DSNP v2.1.

(C) Genes at the 22q11.21 locus. Genes shown in red have been analysed in Figure 2.

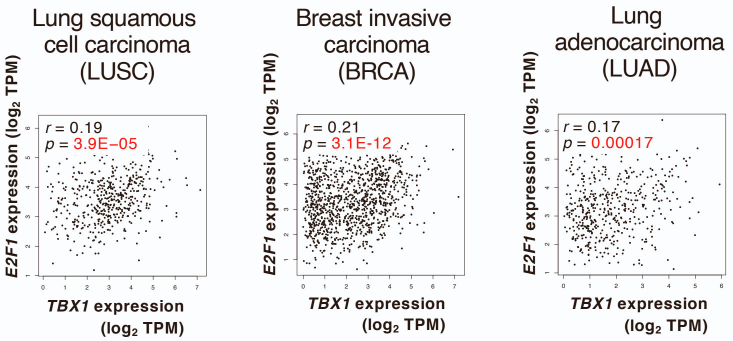

Figure S7. *E2F1* expression levels are positively correlated with *TBX1* expression levels.
Pearson correlations (r) between *E2F1* and *TBX1* expression levels in The Cancer Genome Atlas database.

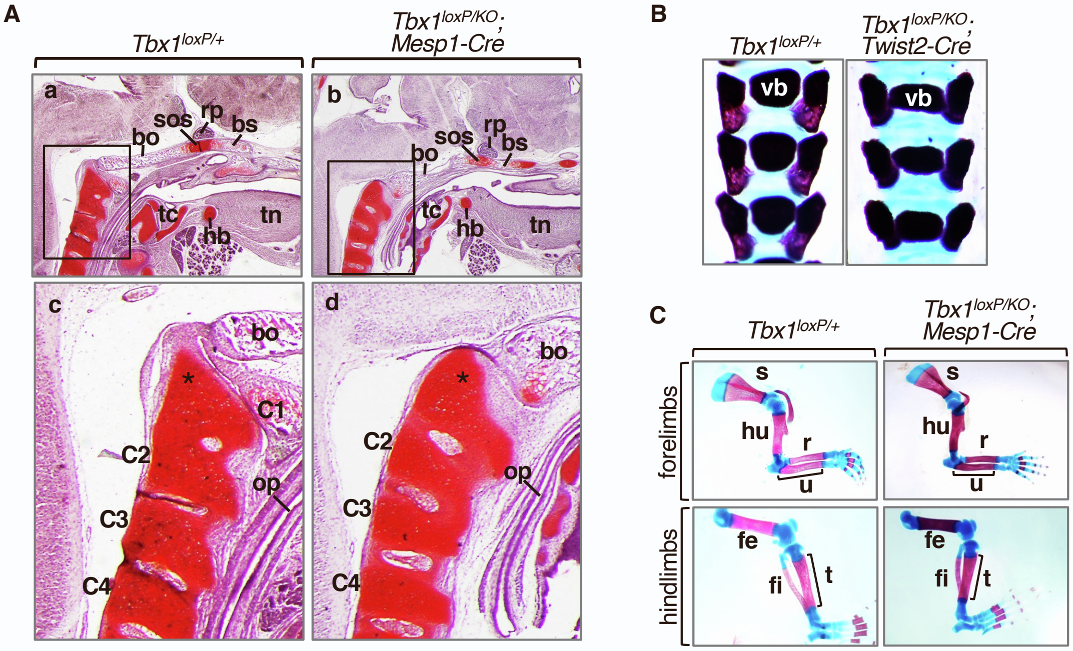

Figure S8. ***Tbx1$^{loxP/KO}$;Mesp1-Cre*** **and** ***Tbx1$^{loxP/KO}$;Twist2-Cre*** **mice recapitulate the phenotypes of** ***Tbx1*****-knockout mice.**

(A) Sagittal sections of E16.5 control (a,c) and *Tbx1$^{loxP/KO}$;Mesp1-Cre* (b,d) embryos were stained with safranin O/hematoxylin/Fast Green and observed at low (a,b) and high (c,d) magnification. Asterisk, the odontoid process of C2. bo, the primordium of the basioccipital bone; bs, the primordium of the basisphenoid bone; sos, spheno-occipital synchondrosis; rp, Rathke's pouch; hb, hyoid bone; tc, thyroid cartilage; tn, tongue; op, oropharynx.

(B) Ventral view of lumbar vertebrae (L4–L6) of control and *Tbx1$^{loxP/KO}$;Twist2-Cre* neonates stained for bone and cartilage. vb, vertebral body.

(C) Bone staining of forelimbs and hindlimbs of control and *Tbx1$^{loxP/KO}$;Mesp1-Cre* neonates. Brackets indicate the ossified shaft of the ulna (u) and tibia (t). s, scapula; hu, humerus; r, radius; fe, femur; fi, fibula.

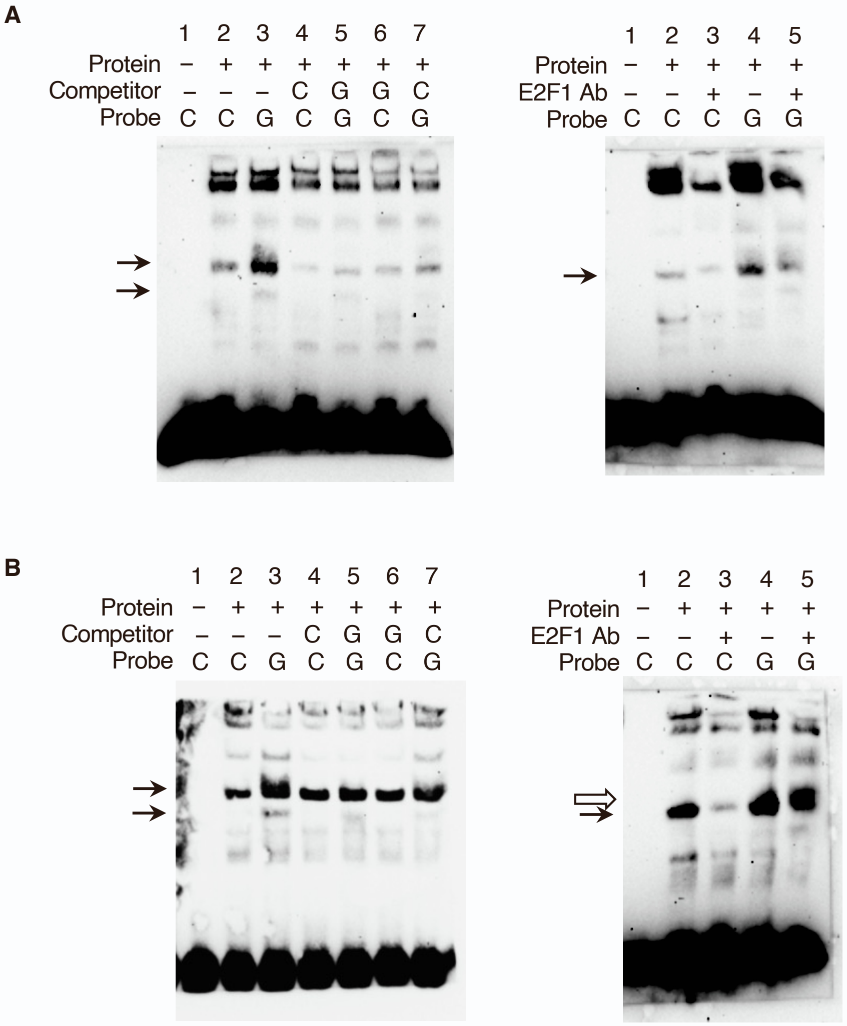

Figure S9. Full-length images of representative electrophoretic mobility shift assays related to Figure 3D. (A,B) Electrophoretic mobility shift assays (EMSAs) with biotin-labelled probes containing rs41298798-[C] or rs41298798-[G] alleles in E2F1-transfected COS1 (A) and HeLa cells (B). Black arrows, allele-specific bands that interact with nuclear proteins; white arrow, a super-shifted E2F1 complex.

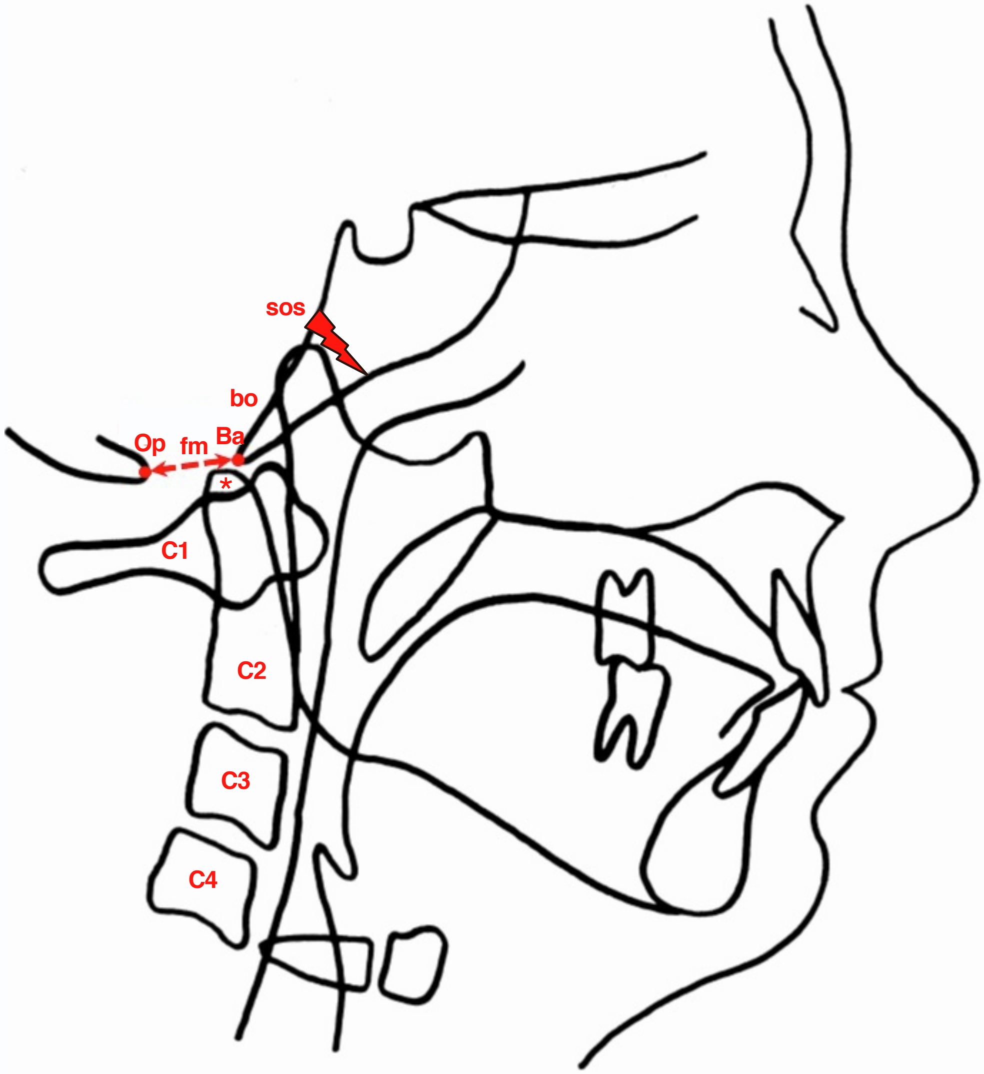

Figure S10. Cephalometric landmarks used in this study.
Ba: Basion, the lowest point on the anterior border of the foramen magnum (fm); Op: Opisthion, the posterior border of the foramen magnum; bo, basioccipital bone; sos, spheno-occipital synchondrosis; asterisk, C2 odontoid process.

Supplemental Methods

A list of mouse genes associated with abnormal basicranium morphologies.

Basicranium morphology	Gene
Abnormal basicranium angle	*Bmp7 Smg1*
Abnormal foramen magnum morphology	*Ank Fgfr3 Fgfrl1 Foxc1 Idua Ltbp3 Pth1r Tbx15 Tbx1*
Abnormal basioccipital bone morphology	*Ank Arl6 Baiap2 Bmp7 Cdx1 Chrd Ctnnb1 Dlx5 Dyrk2 Evc Fgfr2 Fgfrl1 Foxc2 Foxd3 Foxn3 Gli2 Hhat Hoxa1 Hoxa2 Hoxa3 Hoxd4 Ltbp1 Ltbp3 Memo1 Nedd4 Nkx3-2 Pcgf2 Pgap1 Psen1 Rara Rarb Rarg Rpgrip1l Rr45 Sh3pxd2a Ski Tbx1 Tbx15 Tmem107 Wnt9a*
Abnormal basisphenoid bone morphology	*Adamts3 Alx1 Alx4 Ankrd11 Apaf1 Arl6 Baiap2 Bmp7 Bmper Boc Brd2 Cdon Chrd Coro1c Crkl Ctnnb1 Dhx35 Dicer1 Disp1 Dlx2 Dlx5 Dlx6 Dyrk2 Msx1 Snrpb Ednra Evc Evc2 Eya1 Fgfr1 Fgfr2 Fgfrl1 Foxc1 Foxc2 Foxn3 Gas1 Gas2l2 Shh Gli2 Gli3 Gm5544 Kmt2d Memo1 Srsf3 Hand1 Hmgxb3 Hoxa2 Kif3a Ltbp1 Ltbp3 Memo1 Men1 Mn1 Morc2a Nkx3-2 Nosip Nsun2 Otx1 Otx2 Pdgfra Pkdcc Prtg Ptch1 Ptprf Ptprs Rara Rarg Rpgrip1l Rr45 Rr115 Shh Runx2 Sh3pxd2a Ski Smg9 Smo Sox9 Sp3 Ssr2 Stk11 Sulf1 Sulf2 Tbx1 Tcf7l2 Tmem107 Trps1 Wnt1 Wnt3a Zeb1*
Abnormal presphenoid bone morphology	*Alx1 Alx4 Apaf1 Arid5b Arl6 Bmp2 Chrd Chuk Csrnp1 Dicer1 Dlx5 Dlx6 Dyrk2 Fgfr2 Fgfr3 Foxc2 Foxf2 Gli2 Gsc Kmt2d Memo1 Irf6 Lmo4 Mks1 Mn1 Otx1 Otx2 Phc1 Phc2 Pkd1 Ptch1 Rara Rarg Rr133 Schip1 Sgpl1 Ski Smo Sox9 Sp3 Stk11 Tiparp Tmem107 Trps1 Vax1 Wnt1 Wnt3a Zfp640 Zfp950*
Abnormal spheno-occipital synchondrosis (SOS)	*Arl6 Csgalnact1 Fgfr2 Pkd1 Tbx1 Chrd Ctnnb1 Fgfrl1 Ihh Mef2c Pth1r Six1 Six4 Chrd Por Nppc Twist1 Lef1 Pthlh Runx2 Six2 Kif3a Hdac4 Map2k1 Id2 Ift88 Alpl Ltbp3 Fgfr3*
Abnormal intersphenoid synchondrosis (ISS)	*Arl6 Evc Evc2 Fgfr2 Pkd1 Pkd2 Lef1 Ctnnb1 Pthlh Runx2 Six2 Kif3a Hdac4 Map2k1 Id2 Ift88 Alpl Ltbp3 Fgfr3*
Short basicranium	*Acan Arl6 B3glct Bcl11b Bmp7 Fgfr2 Fgfr3 Foxc1 Hapln1 Ltbp3 Mbtps1 Rr23 Rr25 Src*
Premature cranial synchondrosis closure	*Fgfr3 Pfas Pkd1*

A list of mouse genes associated with "abnormal basicranium morphologies" was obtained from the Mouse Genome Informatics (MGI) database[5] and the published review.[6] See Table S5 for full information on the MGI database.

Sequences for single-guide RNA cloning and validation.

sgRNA	Sequence	PAM
5′ Δ41 Guide 1	5′-CCGATTTGACCGGTAGACAA-3'	5′-AGG-3'
3′ Δ41 Guide 2	5′-TGGCTGTTCGGACTCCGAGC-3'	5′-TGG-3'
Precise editing	5′-TGGCTGTTCGGACTCCGAGC-3'	5′-TGG-3'
**Genotyping primers**	**Sequence**	
Left primer	5′-TAAAGACCAAGAGACAAGGGGA-3'	
Right primer	5′-AAACAGAAACCACGAGAAGGCT-3'	
**ssODN**	**Sequence**	
ssODN-C	5′-caagttttgcagatgcacccgatttgaccggtagacaaaggcgggtgccgCgctgtgtctaatgtacacaccagctcg gagtccgaacagccaaggggagc-3'	
ssODN-G	5′-caagttttgcagatgcacccgatttgaccggtagacaaaggcgggtgccgGgctgtgtctaatgtacacaccagctcg gagtccgaacagccaaggggagc-3'	

Sequences of primers used for quantitative polymerase chain reaction.

Gene	Sense Sequence	Antisense Sequence
TBX1	5'-TAGCGAGAAATATGCCGAGGA-3'	5'-CGTGATCCGATGGTTCTGGT-3'
RANBP1	5'-AATACAGACGAGTCCAACCATGA-3'	5'-GAACAGTTTTGCCCGCATTTTA-3'
GNB1L	5'-CGGCTATGAGGATGGATCG-3'	5'-CTGGGAGTCAAAGTCAAGGTC-3'
TANGO2	5'-CTGGCAGCACTCACCAACTAC-3'	5'-GTCAGTGGTCAGAAAGTGGGT-3'
COMT	5'-GAAGGGGACAGTGCTACTGG-3'	5'-CAGGAACGATTGGTAGTGTGTG-3'
ARVCF	5'-CTATTGTCACATCCGAAGATGGC-3'	5'-CGTACTGTCCGAGTGGTCAC-3'
TXNRD2	5'-CTAGCCCCGACACTCAGAAGA-3'	5'-GGCCATGATCGCTATGGGT-3'
RTL10	5'-GTTTTGCTGAGTACCATGCTGT-3'	5'-GCCAGGCCCTCTAAGAACC-3'
SEPTIN5	5'-CGCATCAGCCAGACGGTAG-3'	5'-CCGCTCTCATCACGGAAGT-3'
DGCR8	5'-GCAGAGGTAATGGACGTTGG-3'	5'-AGAGAAGCTCCGTAGAAGTTGAA-3'
CRKL	5'-CTGTCGGTGTCCGAGAACTC-3'	5'-TGGTCCCCGATCTTAAAACGG-3'
GAPDH	5'-GACAGTCAGCCGCATCTTCT-3'	5'-GCGCCCAATACGACCAAATC-3'

Supplemental References

1. Peyregne, S., Boyle, M.J., Dannemann, M., and Prufer, K. (2017). Detecting ancient positive selection in humans using extended lineage sorting. Genome Res. *27*, 1563–1572. 10.1101/gr.219493.116.
2. Kuhlwilm, M., and Boeckx, C. (2019). A catalog of single nucleotide changes distinguishing modern humans from archaic hominins. Sci. Rep. *9*, 8463. 10.1038/s41598-019-44877-x.
3. Kou, I., Otomo, N., Takeda, K., Momozawa, Y., Lu, H.-F., Kubo, M., Kamatani, Y., Ogura, Y., Takahashi, Y., Nakajima, M., et al. (2019). Genome-wide association study identifies 14 previously unreported susceptibility loci for adolescent idiopathic scoliosis in Japanese. Nat. Commun. *10*, 3685. 10.1038/s41467-019-11596-w.
4. Yang, D., Jang, I., Choi, J., Kim, M.S., Lee, A.J., Kim, H., Eom, J., Kim, D., Jung, I., and Lee, B. (2018). 3DIV: A 3D-genome Interaction Viewer and database. Nucleic Acids Res. *46*, D52–D57. 10.1093/nar/gkx1017.
5. Blake, J.A., Baldarelli, R., Kadin, J.A., Richardson, J.E., Smith, C.L., Bult, C.J., and Mouse Genome Database Group (2021). Mouse Genome Database (MGD): Knowledgebase for mouse-human comparative biology. Nucleic Acids Res. *49*, D981–D987. 10.1093/nar/gkaa1083.
6. Funato, N. (2020). New insights into cranial synchondrosis development: A mini review. Front. Cell Dev. Biol. *8*, 706. 10.3389/fcell.2020.00706.
