# Supplementary material for: A regulatory variant impacting TBX1 expression contributes to basicranial morphology in Homo sapiens
Source: Am J Hum Genet. 2024 Apr 11;111(5):939–53. doi: 10.1016/j.ajhg.2024.03.012 (PMC11080286; doi:10.1016/j.ajhg.2024.03.012)
Supplement: Document S2. Article plus supplemental information [file mmc3.pdf]

A regulatory variant impacting *TBX1* expression contributes to basicranial morphology in *Homo sapiens*

Graphical abstract

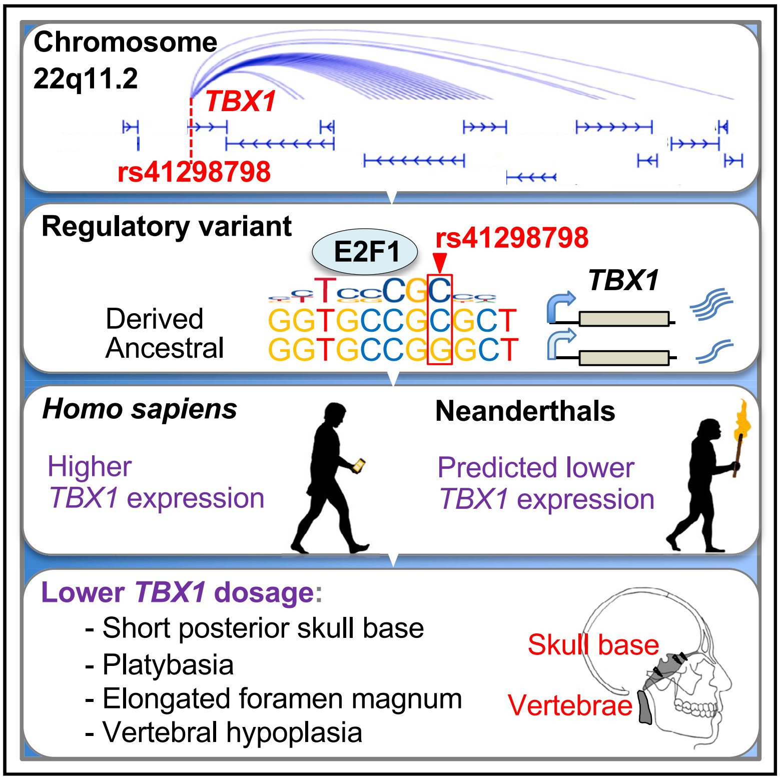

Authors

Noriko Funato, Arja Heliövaara, Cedric Boeckx

Correspondence

noriko-funato@umin.ac.jp

Changes in gene regulatory elements are crucial for human phenotypic divergence. However, identifying the changes responsible for the distinct morphology of *Homo sapiens* remains challenging. Here, we report a noncoding SNP, rs41298798, in *TBX1* as a potential causal variant contributing to the basicranial morphology found in *Homo sapiens*.

Funato et al., 2024, The American Journal of Human Genetics *111*, 939–953
May 2, 2024 © 2024 The Author(s).
https://doi.org/10.1016/j.ajhg.2024.03.012

CellPress

ARTICLE

A regulatory variant impacting *TBX1* expression contributes to basicranial morphology in *Homo sapiens*

Noriko Funato,[1,2,*] Arja Heliövaara,[3] and Cedric Boeckx[4,5,6,7]

Summary

Changes in gene regulatory elements play critical roles in human phenotypic divergence. However, identifying the base-pair changes responsible for the distinctive morphology of *Homo sapiens* remains challenging. Here, we report a noncoding single-nucleotide polymorphism (SNP), rs41298798, as a potential causal variant contributing to the morphology of the skull base and vertebral structures found in *Homo sapiens*. Screening for differentially regulated genes between *Homo sapiens* and extinct relatives revealed 13 candidate genes associated with basicranial development, with *TBX1*, implicated in DiGeorge syndrome, playing a pivotal role. Epigenetic markers and *in silico* analyses prioritized rs41298798 within a *TBX1* intron for functional validation. CRISPR editing revealed that the 41-base-pair region surrounding rs41298798 modulates gene expression at 22q11.21. The derived allele of rs41298798 acts as an allele-specific enhancer mediated by E2F1, resulting in increased *TBX1* expression levels compared to the ancestral allele. *Tbx1*-knockout mice exhibited skull base and vertebral abnormalities similar to those seen in DiGeorge syndrome. Phenotypic differences associated with *TBX1* deficiency are observed between *Homo sapiens* and Neanderthals (*Homo neanderthalensis*). In conclusion, the regulatory divergence of *TBX1* contributes to the formation of skull base and vertebral structures found in *Homo sapiens*.

Introduction

Genetic variants that distinguish *Homo sapiens* from closely related extinct hominins, for whom high-coverage genomes are available, are predominantly located in the noncoding regions of the genome.[1–4] These noncoding variants, particularly in regulatory regions, have the potential to affect gene expression.[5–7] Changes in this regulatory program are likely to have had a significant impact on human evolution, with evidence suggesting that these changes underlie morphological differences between our closest relatives.[2,8–10] Noncoding single-nucleotide polymorphisms (SNPs) often affect gene expression by altering the function of enhancer elements and are under evolutionary pressure.[6,7,11,12] In addition, these noncoding SNPs have also been implicated in human disease by playing a critical role in controlling the expression of target genes during development,[13] although most noncoding variants associated with disease susceptibility are unlikely to be strongly deleterious.[14] To improve our understanding of the genetic and molecular basis of morphological differences in *Homo sapiens*, the identification of causal variants and the interpretation of the biological impact of regulatory divergence on human evolution are essential.[9,15] However, pinpointing these causal variants remains extremely challenging.[16]

The skull of *Homo sapiens* has acquired unique cranial features among primates, including a highly flexed skull base, with an increase in absolute and relative brain size during hominin evolution.[17,18] Compared to modern humans (*Homo sapiens*), closely related extinct hominins and other great apes have different skull base phenotypes, including a flatter basicranium, a shorter length of the posterior skull base, and an anteroposteriorly elongated foramen magnum.[16,17,19,20] It is hypothesized that the distinctive morphology and evolution of the human skull are influenced, at least in part, by changes in brain development and embryonic brain-skull interactions.[18,21] The synchondroses of the skull base play a critical role in embryonic and postnatal skull elongation.[22] To retain their capacity for accelerated fetal and postnatal growth, synchondroses must remain unmineralized as cartilage.[22,23] In particular, the spheno-occipital synchondrosis (SOS) persists in the endochondral basicranium and does not ossify until 16–18 years of age in humans, contributing to the longitudinal growth of the skull.[22] In the vertebral column, dysmorphic vertebrae and platyspondyly are frequently observed in our closest extinct relatives.[24,25]

Studying the mechanisms driving human diseases and pathological conditions in skeletal morphology can provide clues to evolutionary anatomical changes.[16] Chromosome 22q11.2 deletion is one of the most common genetic microdeletions in humans.[26] A 1.5 Mb hemizygous deletion of 22q11.2 causes most craniofacial phenotypes of DiGeorge syndrome (DGS [MIM: 188400]) and velocardiofacial syndrome (VCFS [MIM: 192430]). *TBX1* (MIM: 602054), located

[1]Department of Signal Gene Regulation, Graduate School of Medical and Dental Sciences, Tokyo Medical and Dental University (TMDU), Yushima 1-5-45, Bunkyo-ku 113-8510, Tokyo, Japan; [2]Research Core, Tokyo Medical and Dental University (TMDU), Yushima 1-5-45, Bunkyo-ku 113-8510, Tokyo, Japan; [3]Cleft Palate and Craniofacial Center, Department of Plastic Surgery, Helsinki University Hospital and Helsinki University, Stenbäckinkatu 11, P.O. Box 281, Helsinki FI-00029 HUS, Finland; [4]Catalan Institute for Advanced Studies and Research (ICREA), Passeig de Lluís Companys, 23, 08010 Barcelona, Spain; [5]Section of General Linguistics, University of Barcelona, Gran Via de les Corts Catalanes 585, 08007 Barcelona, Spain; [6]University of Barcelona Institute for Complex Systems, Gran Via de les Corts Catalanes 585, 08007 Barcelona, Spain; [7]University of Barcelona Institute of Neurosciences, Gran Via de les Corts Catalanes 585, 08007 Barcelona, Spain
*Correspondence: noriko-funato@umin.ac.jp
https://doi.org/10.1016/j.ajhg.2024.03.012.
© 2024 The Author(s). This is an open access article under the CC BY license (http://creativecommons.org/licenses/by/4.0/).

at 22q11.21, encodes T-box transcription factor 1. Heterozygous loss-of-function mutations in *TBX1* also cause DGS, VCFS, and conotruncal anomaly face syndrome (MIM: 217095).[26–29] Some individuals with DGS/VCFS show changes in the structure of the skull base and the vertebral column.[30–34] *Tbx1* (GenBank: 21380) knockout (KO) mice exhibit cardiac and craniofacial phenotypes that mirror those observed in individuals with DGS/VCFS.[35–38] During mouse embryonic development, TBX1 is localized in the cartilaginous primordium of the posterior skull base and plays a critical role in maintaining the undifferentiated phenotype of chondroprogenitors in the SOS.[39] In *Tbx1*-KO mice, the SOS in the skull base is completely mineralized at birth,[39] and the anterior arch of the first cervical vertebra is aplastic.[35,37,38] Using the similar skeletal phenotypes of *Tbx1*-KO mice and DGS/VCFS to investigate the morphological effects of *Tbx1* and *TBX1* dosage may provide a basis for understanding morphological changes in modern human lineage.

In the present study, we identified an ancestral allele within the *TBX1* locus that may contribute to the basicranial morphology found in *Homo sapiens*. To elucidate how the *TBX1* locus influences basicranial morphology, we identified the target genes regulated by SNP rs41298798 and the mechanism by which this SNP controls *TBX1* expression. Furthermore, we analyzed the effects of *TBX1* dosage on the basicranial morphology found in *Homo sapiens*.

Material and methods

Bioinformatic analysis

We identified mouse genes associated with annotated anatomical abnormalities in the skull base from the Mouse Genome Informatics (MGI) (accessed on July 31, 2023) and PubMed databases (supplemental methods). The allele frequencies of the SNPs were compiled from the 1000 Genomes Project dataset.[40] To characterize and obtain functional annotations of ancestral alleles at the 22q11.21 locus, we used combined annotation-dependent depletion (CADD) v1.6,[41] HaploReg v4.2,[42] 3DSNP v2.0,[43] and RegulomeDB v2.0.3.[44] Histone markers in the 22q11.21 locus were queried using Encyclopedia of DNA Elements (ENCODE) phase 3[45] across mesenchymal stem cells and MG63 cells. Reads per million (rpm)/base-pair (bp) plots were generated for H3K27ac, H3K4me3, H3K36me3, H3K27me3, and H3K9me3 using the UCSC Genome Browser. To identify candidate causal genome-wide association study (GWAS) variants that alter gene expression, we searched for available the expression quantitative trait locus (eQTL) data using the Genotype-Tissue Expression (GTEx) project.[46] The eQTL data are summarized in Table S1. We visualized and intersected variants with chromatin annotations in H1 mesendoderm cells[47] using the 3D Interaction Viewer (3DIV).[48] The mRNA expression data for normal tissues and tumors were obtained from the Gene Expression Profiling Interactive Analysis (GEPIA)[49] web application using The Cancer Genome Atlas (TCGA) database.[50] The correlation of mRNA-mRNA pairs of the gene set from human tumors was analyzed by calculating the Pearson's correlation coefficient.

The differential effect of rs41298798 alleles on transcription factor (TF) binding was predicted for all human TF motif sets using JASPAR[51] with a relative score >0.85 as the threshold for significance. Twenty base pairs surrounding rs41298798 were evaluated, and the putative TF-binding motifs for rs41298798-C and -G were compared.

CRISPR/Cas9 guide selection and genome editing

Genome editing experiments were performed using MG63 cells to identify the target genes of rs41298798. Using the CRISPR design tool CHOPCHOP,[52] we selected single-guide RNA (sgRNA) sequences within 100 bp of rs41298798. Annealed oligomers, including guide RNA sequences, were subcloned into pSpCas9(BB)-2A-GFP (PX458) or pSpCas9(BB)-2A-Puro (PX459) V2.0 (plasmids #48138 and #62988; Addgene, Watertown, MA, USA; gifted by Feng Zhang)[53] containing expression cassettes for the guide RNA and human-codon-optimized Cas9. Plasmids were transformed into chemically competent *E. coli* (DH5-alpha), and after culturing the cells, the plasmid DNA was extracted and purified. MG63 cells (TKG0294; the Cell Resource Center for Biomedical Research, Tohoku University, Sendai, Japan) were routinely grown in Dulbecco's modified Eagle's medium (DMEM) supplemented with 10% fetal bovine serum (FBS) and 1% antibiotics (100 U/mL penicillin and 0.1 mg/mL streptomycin) until transfection.

To delete 41 bp of noncoding sequence around rs41298798, we employed a dual-guide RNA strategy using two Cas9-guide RNA constructs with a 29-bp spacing between them. We plated MG63 cells in 24-well plates and co-transfected with 250 ng of each CRISPR construct using Lipofectamine 2000 (Thermo Fisher Scientific, Waltham, MA, USA). Clones with genomic deletions were screened using agarose gel electrophoresis of the PCR amplicons. The Δ41/Δ41 clones were expanded along with the wild-type clones, which were also exposed to the CRISPR/Cas9 complex. In each cell line, three wild-type and three biallelic 41 bp deletions were selected for further study. To generate isogenic MG63 cells that were either C/C or G/G at rs41298798, we plated MG63 cells in 12-well plates and transfected 500 ng of CRISPR plasmid constructs and 313 ng of a 100 bp single-stranded oligodeoxynucleotide donor template containing either the C or G allele. After transfection, the MG63 cells were selected using 0.25 μg/mL puromycin for 5 days. After cell growth, single colonies were isolated and genomic DNA was extracted. A 163-bp region flanking rs41298798 was PCR amplified, purified, and genotyped at rs41298798 using restriction fragment-length polymorphism assays. The purified product was digested with HhaI and electrophoresed on a 2% agarose gel, and the cleavage patterns were qualitatively analyzed. To account for off-target effects of the Cas9 nuclease, we selected three derived rs41298798-C/C and three ancestral rs41298798-G/G clones for further study. The primer sequences used for genome editing are listed in supplemental methods.

Quantitative PCR (qPCR) analysis

Total RNA was extracted from cultured cells using TRIzol (Thermo Fisher Scientific) and an RNeasy Mini Kit (Qiagen, Hilden, Germany). mRNA analysis was performed using SuperScript IV VILO Master Mix (Thermo Fisher Scientific) and PowerUP SYBR Green PCR Master Mix (Thermo Fisher Scientific). The amplification and detection of mRNAs were performed using the StepOnePlus Real-Time PCR System (Thermo Fisher Scientific). mRNA

expression levels were normalized to *GAPDH* (GenBank: 2597) levels. The relative quantity was calculated using the $2^{-\Delta\Delta Ct}$ method.[54] All qPCR assays were performed in duplicate in at least three independent experiments using three different samples. The primer sequences used for qPCR are listed in supplemental methods.

Luciferase assay

Luciferase reporter vectors were constructed by cloning the *TBX1* promoter (−912/+63; sense: 5′-GTTGGTACCCTCCTCAGTGCTTC CCTTTG-3′ and antisense: 5′-ACTCTCGAGAGTGTTCCTCCCTCC CTCAC-3′) with or without oligonucleotides (sense: 5′-AGGCG GGTGCCGSGCTGTGTCTAAT-3′ and antisense: 5′-ATTAGACACA GCSCGGCACCCGCCT-3′) containing either derived or ancestral alleles of rs41298798 into the pGL2-Basic vector (Promega, Madison, WI, USA). The *E2F1* (MIM: 189971) expression vector has been described previously.[55]

MG63 and COS1 cells (RCB0143; RIKEN Cell Bank, Tsukuba, Japan) were cultured in DMEM supplemented with 10% FBS and 1% antibiotics. The cells were seeded into 24-well plates at 1×10^5 cells/well. The cells were transfected with 250 ng of the pGL2 constructs with 50 ng of a *lacZ* (GenBank: 945006) expression vector using TransFectin reagent (Bio-Rad Laboratories, Hercules, CA, USA). Cell lysates were harvested after 48 h and assayed on a FLUOstar OPTIMA-6 instrument (BMG Labtech, Ortenberg, Germany) using a Luciferase Reporter Assay System (Promega) according to the manufacturer's instructions.

Electrophoretic mobility shift assays (EMSAs)

We prepared probes for the derived (C) and ancestral (G) alleles of rs41298798 by annealing 25-bp complementary oligonucleotides (sense: 5′-AGGCGGGTGCCGSGCTGTGTCTAAT-3′ and antisense: 5′-ATTAGACACAGCSCGGCACCCGCCT-3′) and labeling them using a biotin 3′ End DNA Labeling Kit (Thermo Fisher Scientific). Nuclear proteins were isolated from *E2F1*-overexpressing COS1 and HeLa cells (TKG 0331; Cell Resource Center for Biomedical Research). DNA-protein binding reactions were performed using a LightShift Chemiluminescent EMSA kit (Thermo Fisher Scientific) according to the manufacturer's instructions. For competition assays, nuclear proteins were pre-incubated with excess unlabeled probes before adding biotin 3′ end-labeled probes in band shift buffer (10 mM Tris-HCl [pH 7.5], 100 mM KCl, 10 mM EDTA, 2.5% glycerol, 50 ng/μL of poly(dI-dC)). For supershift assays, 2 μg of anti-E2F1 antibody (sc-251X; Santa Cruz Biotechnology, Dallas TX, USA) was added to the reaction mixture and it was incubated for 30 min at room temperature. The binding reaction mixtures were separated by electrophoresis on a 4.5% polyacrylamide gel in 0.5× Tris-borate-EDTA buffer and transferred onto Hybond-N^+ membranes (Amersham, Stafford, UK). The biotin-labeled DNA was detected using a Chemiluminescent Nucleic Acid Detection Module (Thermo Fisher Scientific). Images of uncropped gels are shown in Figure S9.

Mouse lines

$Tbx1^{tm1Dsr}$ (synonym: $Tbx1^{neo}$; MGI: 3510038; gifted by Deepak Srivastava)[37] has been used for tissue-specific deletion of *Tbx1* in mice.[38] ICR.Cg-$Mesp1^{tm2(cre)Ysa}$/YsaRbrc (hereafter referred to as *Mesp1-Cre*; stock no. RBRC01145, RIKEN)[56] and B6.129X1-$Twist2^{tm1.1(cre)Dor/}$J (hereafter referred to as *Twist2-Cre*; stock no. 008712; Jackson Laboratory, Bar Harbor, ME, USA)[57] mice have been described previously. Heterozygous mice ($Tbx1^{loxP/+}$)[38] were mated with $Meox2^{tm1(cre)Sor}$ (also known as *More-Cre* mice; gifted by Michelle Tallquist),[58] resulting in the heterozygous *Tbx1* null allele ($Tbx1^{KO/+}$).[38] Subsequently, $Tbx1^{tm1.1Dsr}$ (synonym: $Tbx1^{null}$; MGI: 3510040; *Tbx1*-KO mice; mixed genetic strain background) were generated in which the gene is knocked out in all tissues.[37,38] Wild-type and $Tbx1^{loxP/+}$ littermates were used as controls. All experimental animal procedures were reviewed and approved by the Institutional Animal Care and Use Committee of the Tokyo Medical and Dental University (permit number 0126215C, February 24, 2016). All experiments and methods were performed in accordance with relevant guidelines and regulations.

Micro-computed tomography (micro-CT)

Mineralized tissue formation of *Tbx1*-KO neonates was assessed using micro-CT. Images were scanned at a voltage of 100 kV and 30 μA in beam current using an inspeXio SMX-100CT instrument (Shimadzu, Kyoto, Japan) at a pixel size of 512 × 512 and voxel size of 0.049 mm/voxel. The results were further analyzed using a TRI-3D-BON imaging system (Ratoc, Santa Clara, CA, USA). 3D images were rotated at specific angles to generate sagittal and bird's-eye views of the skull base.

Bone staining and histology

For bone staining, *Tbx1*-KO, $Tbx1^{loxP/KO}$;*Twist2-Cre*, and $Tbx1^{loxP/KO}$;*Mesp1-Cre* neonates were harvested and fixed in 95% ethanol. Bones were stained with alizarin red and Alcian blue to detect mineralized and cartilaginous regions, respectively. For histology, *Tbx1*-KO and $Tbx1^{loxP/KO}$;*Mesp1-Cre* mouse embryos were harvested and fixed in 4% paraformaldehyde at 4°C overnight. Paraffin-embedded sections were stained with safranin O/haematoxylin/Fast Green to detect cartilage.

Cephalometric analysis

Lateral cephalometric radiographs were obtained to record the cephalometric values in the clinical records for orthodontic diagnosis and treatment. Cephalometric values of children with DGS/VCFS (22q11.2 deletion syndrome; mean age 8.5 years, range 5.8–12.9 years, both sexes) were compared with the values of 41 healthy age- and sex-matched controls, as previously reported.[34] The research protocol was approved by the Helsinki University Hospital (HUS/234/2020 §57, December 22, 2020) and adhered to the principles outlined in the Declaration of Helsinki. In accordance with the Medical Research Act (Ministry of Social Affairs and Health, Finland), ethical approval was not required for the retrospective archival cephalometric study. The Register and Privacy Statement was formulated and approved in accordance with the European Union (EU) General Data Protection Regulation to ensure secure data protection.

Statistics

Experiments were performed on at least three independent occasions and the results are presented as the mean ± standard error of the mean for *n* experiments. Data were analyzed using PRISM software (version 9.0; GraphPad, San Diego, CA, USA) or Microsoft Excel. Unpaired or paired two-tailed Student's t tests were used to compare two groups of independent samples. One-way analysis of variance (ANOVA) with Dunnett's post-hoc test was used to analyze the differences among three or more groups. A two-way ANOVA with Sidak's multiple comparison test was performed to compare the transcriptional activity between genotypes and in

response to *E2F1* overexpression. We used binomial tests to compare phenotypes across groups where success was defined as a match in the phenotypes between two pairs, with 50% concordance expected by chance. We compared differences between *Tbx1*-KO and wild-type mice to differences between modern humans and Neanderthals. Similarly, we compared how phenotypes differed between individuals with DGS/VCFS and unaffected individuals to differences found between modern humans and Neanderthals. The overlapping phenotypes shown in detail in Table S4 are summarized in Table 2. Statistical significance is presented as $*p < 0.05$, $**p < 0.01$, $***p < 0.001$, and $****p < 0.0001$.

Results

Prioritization of candidate genes and variants

To identify genes that may be involved in the development of the skull base in modern humans, we screened putative positively selected genes that may be differentially regulated at the skull base between *Homo sapiens* and other hominins using a list of predicted target genes of ancestral alleles that underwent positive selection on the human lineage,[4] a list of human genes with human-lineage high-frequency missense changes,[3] and a list of mouse genes associated with "abnormal basicranium morphologies" from the MGI and PubMed databases (Figure 1A). Thirteen genes (*EVC2*, *TBX1*, *DISP1*, *GLI3*, *OTX1*, *SP3*, *TBX15*, *BCL11B*, *DYRK2*, *TRPS1*, *BRD2*, *HMGXB3*, and *CSGALNACT1*) were annotated as candidate genes that may be differentially regulated at the skull base between *Homo sapiens* and other hominins (Figures 1A and S1A). *EVC2*, encoding EvC ciliary complex subunit 2, is associated with Ellis-van Creveld syndrome (MIM: 225500). *EVC2* has two nucleotide changes distinguishing modern humans from extinct hominins[3] and the regulatory divergence of *EVC2* contributes to the unique craniofacial morphology of the human lineage.[9] Abnormalities have been reported in the frontal region of the skull base of *Evc2*-KO mice.[59] In contrast, *Tbx1* was annotated for abnormalities in the posterior region of the skull base, including an abnormal SOS (Figure S1A). For the ancestral alleles of the seven SNPs at the *TBX1* locus (Table 1), the Neanderthal and Denisova genomes are homozygous for the ancestral alleles.[4] The derived-to-ancestral genotype substitutions were present in chimpanzees and gorillas, whereas they were rare (minor allele frequency [MAF] < 0.02) in modern humans (Tables 1 and S1). These ancestral alleles in the *TBX1* locus were revealed to be more prevalent in South Asians (MAF = 0.041–0.043) and Japanese (MAF = 0.034–0.077) from the 1000 Genomes Project data (Figures S1B and S1C; Tables S1 and S2). The ENCODE project annotates histone marks at genetic loci.[60,61] The introns of *TBX1* were enriched within active histone modification peaks (H3K27ac; Figure 1B), suggesting that the introns of *TBX1* contain functional enhancers. Strong H3K4me3 enhancer signals that overlapped with H3K27ac peaks included SNPs rs41298798, rs72646954, and rs80179718 in seven cell lines (Figure 1B). This element also contains enhancers in stem cells, as indicated by the enrichment of activating marks (H3K4me3 and H3K9ac) and the depletion of H3K9me3 repressive marks (Figure 1B). These data suggest that the introns of *TBX1* contain regulatory elements that may regulate the expression of genes critical for mesenchymal development. Within the region surrounding rs41298798, rs72646954, and rs80179718, a large portion of the sequence was not conserved among mammals (Figure 1B). Algorithms can be used to predict the functional consequences of noncoding SNPs (3DSNP[43]) and to annotate SNPs at a signal (HaploReg[42] and RegulomeDB[44]). To assess the deleterious effects of SNPs, multi-nucleotide substitutions, and insertion/deletion variants, the CADD tool can be used.[41] To identify potential causal SNPs within the *TBX1* locus for functional follow up, we performed *in silico* analyses of these SNPs using CADD, 3DSNP, RegulomeDB, and HaploReg. Among these, rs41298798 was ranked as a promising candidate SNP (Tables 1 and S1). This SNP is associated with positive selection[4] and is the most recent *TBX1*-derived variant based on the framework developed in our previous work.[62] It is predicted to have emerged approximately 300,000 years ago. Based on these results, we selected rs41298798 as the most likely causal SNP contributing to basicranial morphology in *Homo sapiens* at this locus.

The ancestral allele of rs41298798 causes dysregulated expression of genes within the 22q11.21 locus

To identify bone-related cells in which rs41298798 has a relevant regulatory function, we examined the occupancy of histone marks at the locus in cells annotated in the ENCODE database. MG63 osteoblast-like cells expressing *TBX1*[38] showed enrichment of activating methylation marks (H3K4me3) and depletion of repressive marks (H3K27me3 and H3K9me3) at rs41298798 (Figure 2A). To determine whether the expression of genes in the 22q11.21 locus was regulated by a regulatory element present in the *TBX1* intron, we generated MG63 cell lines with homozygous deletions of rs41298798 using CRISPR/Cas9 with flanking sgRNAs (Figure 2B). The sgRNAs were transfected into MG63 cells, and three clones with a bi-allelic 41-bp deletion (Δ41) were generated from the screened clones. The sequence of the 41-bp deletion included rs41298798 and rs1978060 (Figure S2) and putative TF-binding sites (Figure S3). When we examined the expression of genes in the 22q11.21 locus, loss of the 41 bp flanking rs41298798 resulted in higher expression levels of *GNB1L*, *TANGO2*, and *RANBP1* and lower expression levels of *SEPTIN5*, *COMT*, and *DGCR8* compared to their expression levels in wild-type lines (Figure 2C), indicating that the deleted sequence contains a regulatory element. No significant differences in *TBX1* expression levels were observed (Figure 2C).

The association between noncoding SNP genotypes and gene expression levels was assessed using the eQTL approach.[46] To identify the target genes of rs41298798, we searched all available tissue data for eQTL analysis using the GTEx project[46]; however, there were no data for rs41298798 (Table S1). rs1978060 is located 17 bp 3′ of

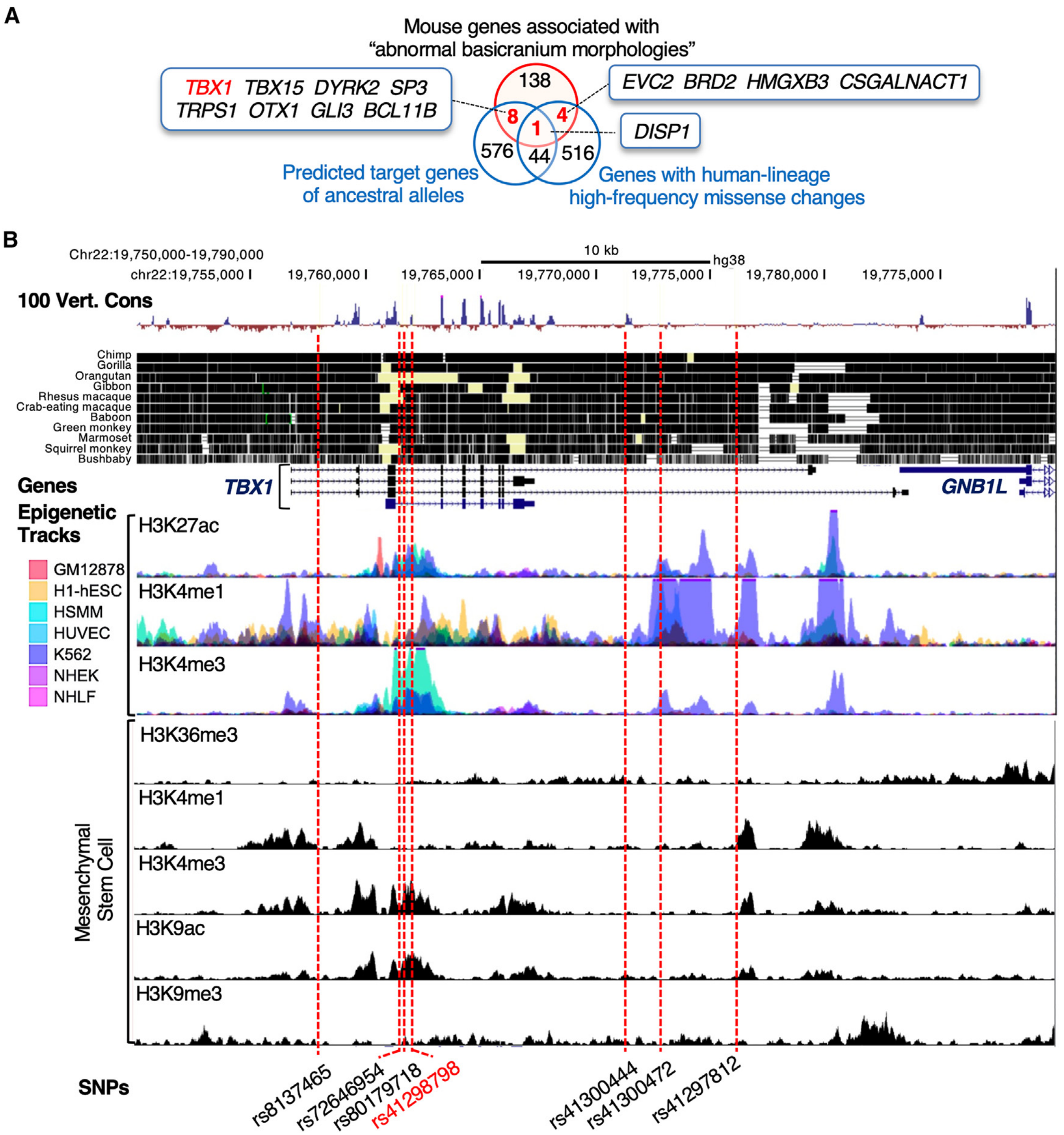

Figure 1. Ancestral alleles are present in the *TBX1* locus

(A) Screening strategy for candidate genes influencing the basicranial morphology of *Homo sapiens*. We used a list of predicted target genes of ancestral alleles,[4] a list of genes with human-lineage high-frequency missense changes,[3] and a list of mouse genes associated with "abnormal basicranium morphologies" obtained from the Mouse Genome Informatics database and PubMed. The list of mouse genes can be found in the supplemental methods and Table S5.

(B) SNPs mapped to introns of the *TBX1*. From top to bottom, the "100 Vert. Cons" track corresponds to sequence conservation across 100 vertebrates, protein-coding genes, epigenetic tracks from the ENCODE database (primary IDs: ENCSR555QHZ, ENCSR196LEI, ENCSR004AKD, ENCSR006GPM, and ENCSR439EHQ), and ancestral alleles. All ENCODE data are plotted as reads per million (rpm)/bp for chromatin immunoprecipitation (ChIP)-sequencing performed on a representative sample of each type.

rs41298798 (Figure S2) and confers a genetic predisposition to adolescent idiopathic scoliosis in the East Asian population.[63] The eQTL analysis showed that rs1978060 was associated with the expression levels of *TBX1*, *GNB1L* (MIM: 610778), and *RTL10* (MIM: 620751; Figure S4). To determine whether the effect on *TBX1* gene expression was mediated by the rs41298798 genotype, we generated isogenic MG63 cell lines with either derived rs41298798-C/C or ancestral rs41298798-G/G genotypes. Three clones of each genotype (C/C and G/G) were selected for expansion (Figure 2D). Of note, qPCR demonstrated a regulatory effect of the genotype at rs41298798 on *GNB1L*, *TANGO2* (MIM: 616830), *RANBP1* (MIM: 601180), *SEPTIN5* (MIM: 602724), *DGCR8* (MIM: 609030), and *COMT* (MIM: 116790), with the ancestral allele driving 59% lower *TBX1* expression levels than the derived allele (Figure 2E; $p = 1.61 \times 10^{-9}$). The expression level of the *CRKL* (MIM: 602007), a potential modifier of cardiac development in 22q11.2 deletion syndrome and a possible target of noncoding putative regulatory

Table 1. Ancestral alleles at variants within the 22q11.21 locus

SNP	Position (GRCh38)	Allele		Population genetics				Scores		
		Ref >Alt	MAF	Derived	Ancestral	Chimp	Gorilla	CADD v1.6	3DSNP v2.0	Regulome v2.0.3
rs8137465	chr22:19757834	T > C	0.016	T	C	C	C	2.829	13.34	0.74401
rs72646954	chr22:19761437	T > C	0.019	T	C	C	C	8.179	37.37	0.60906
rs80179718	chr22:19761655	A > G	0.019	A	G	G	G	7.194	30.52	0.60906
rs41298798	chr22:19761985	C > G	0.017	C	G	G	G	14.530	28.11	0.60906
rs41300444	chr22:19771247	A > G	0.016	A	G	G	G	5.668	2.88	0.60906
rs41300472	chr22:19772783	T > C	0.016	T	C	C	C	5.285	11.86	0.60906
rs41297812	chr22:19776072	C > T	0.018	C	T	T	T	1.465	7.20	0.74401

Ancestral alleles at the *TBX1* locus[4] and *in silico* analyses are shown. Ref, reference allele; Alt, alternative allele; MAF, minor allele frequency; Chimp, chimpanzee.

variants,[64] was unaffected (Figure 2E). Chromatin conformation capture experiments showed that the enhancer region containing rs41298798 physically interacted with the promoters of genes at the 22q11.21 locus in H1 mesendoderm cells (Figure S5). The rs41298798-gene interactions using 3DSNP showed that the *TBX1* is located within three-dimensional (3D) chromatin loops in multiple cell types (Figure S6; Table S3). These data show that the deletion of a small region of putative regulatory DNA at rs41298798 disrupts the normal regulation of genes at the 22q11.21 locus, and that rs41298798 acts as an allele-specific enhancer to modulate the expression of *TBX1*.

E2F1 differentially binds the derived vs. ancestral alleles of rs41298798

Having shown that rs41298798 alters *TBX1* expression levels (Figure 2E), we sought to validate the allele-specific enhancer activity of rs41298798 on the *TBX1* promoter. We constructed luciferase reporter vectors containing the *TBX1* promoter and inserted nucleotides containing either the derived rs41298798-C allele or the ancestral rs41298798-G allele. We then examined the effect of rs41298798 on *TBX1* promoter activity and found that the presence of the rs41298798 allele did not alter *TBX1* promoter activity (Figure 3A). There was no significant difference between the derived allele (C) and the ancestral allele (G; Figure 3A). TFs may be responsible for the allele-specific reporter activity of rs41298798. To test this hypothesis, we searched for TFs that may have differential binding effects on rs41298798. Using JASPAR 2020 CORE,[51] we identified human E2F1 as a candidate TF that could act on the derived rs41298798-C allele (Figure 3B). The ancestral rs41298798-G allele alters a sequence that resembles a consensus E2F1 binding motif (Figure 3B). *E2F1* expression levels were positively correlated with *TBX1* expression levels in samples from the GTEx database (Figure S7). When we overexpressed *E2F1* in MG63 and COS1 cells, the construct containing the derived allele (C) showed higher enhancer activity than the *TBX1* promoter vector with the G allele at rs41298798 (Figure 3C), suggesting that the rs41298798-C allele had enhancer activity against the *TBX1*, which was modulated by E2F1. The ancestral allele (G) significantly reduced E2F1-dependent reporter activity compared to the derived allele (C; Figure 3C).

The allele-specific activity of rs41298798 may be attributed to different binding affinities for E2F1. To experimentally validate the differential binding of E2F1 to the rs41298798 alleles, we performed EMSAs. Consistent with the observed differences in transcriptional activity, the derived and ancestral alleles of rs41298798 showed different DNA-protein complex-binding patterns (Figure 3D). EMSA showed reduced experimental binding in the presence of an anti-E2F1 antibody (Figure 3D). These results provide evidence that rs41298798 acts as an allele-specific enhancer to induce *TBX1* transcriptional activity through E2F1, and they provide an explanation for the association between rs41298798 and changes in *TBX1* expression levels.

Reduced dosage of *Tbx1* contributes to the morphological changes in the posterior skull base and vertebral column

To investigate the effects of *TBX1* that may underlie the morphological changes in our lineage, we analyzed the skeletal phenotypes of *Tbx1*-KO mice to determine whether *Tbx1*-KO phenotypes resembled the divergent phenotypes in our lineage, following an approach pioneered by Gokhman et al.[9] First, we imaged the skull bases of neonatal *Tbx1*-KO mice and their wild-type littermates using micro-computed tomography. Deletion of *Tbx1* in mice resulted in precocious ossification of the SOS and fusion of the basisphenoid and basioccipital bones at the skull base, resulting in a shortened skull base and an anteroposteriorly elongated foramen magnum compared to wild-type littermates (Figure 4A). We also found that *Tbx1*-KO neonates had platybasia (flattening of the skull base) due to inferior displacement of the basioccipital bone at the foramen magnum (Figure 4A).

At the onset of endochondral ossification, the odontoid process (dens) of the second cervical vertebra (C2 or axis) is elongated and projects cranially in the wild-type embryos (Figure 4B). In *Tbx1*-KO embryos, the odontoid process was

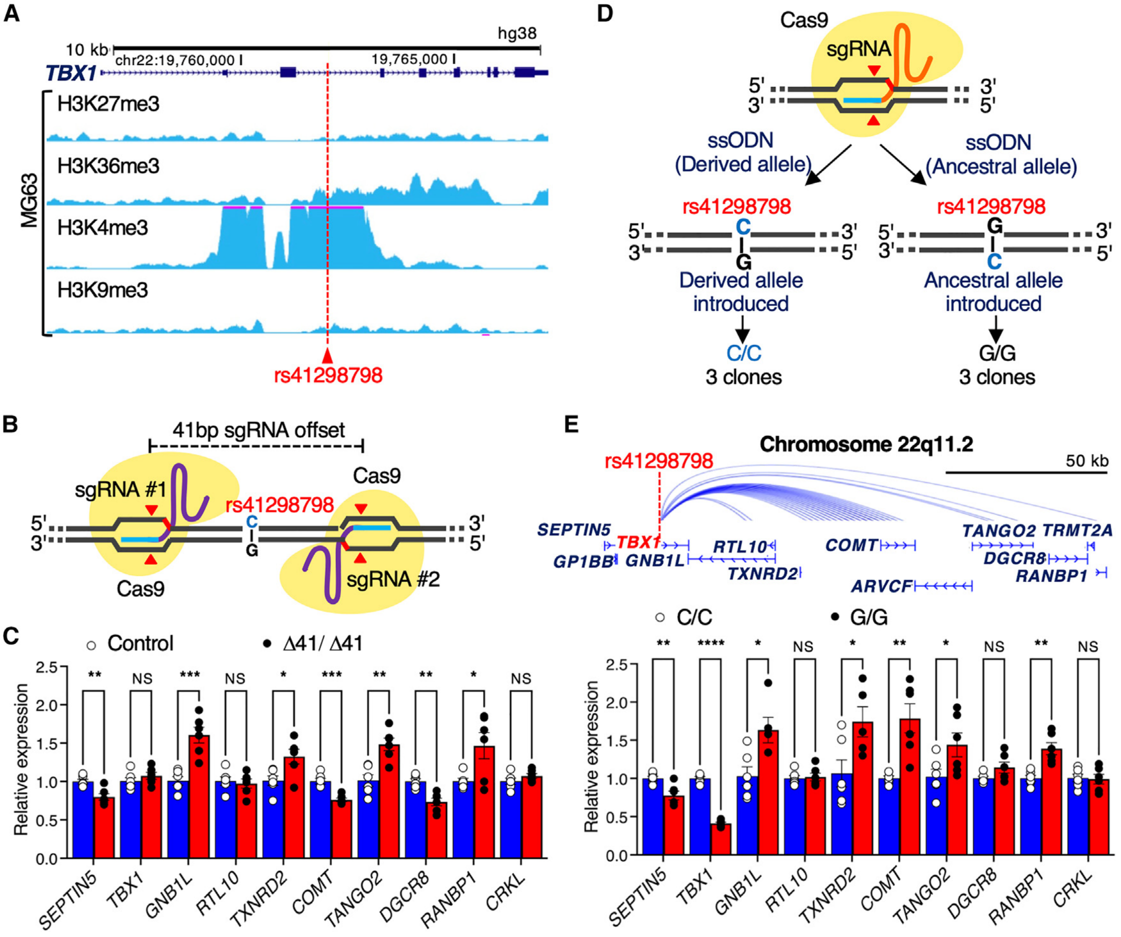

Figure 2. The ancestral allele at rs41298798 causes aberrant expression of genes at the 22q11.21 locus

(A) Epigenetic tracks obtained from the ENCODE database (primary IDs: ENCSR804JFU, ENCSR579SNM, ENCSR380ORO, and ENCSR744EKG). MG63 cells showed enrichment of activating methylation marks (H3K4me3 and H3K36me3) and depletion of repressive marks (H3K27me3 and H3K9me3) at rs41298798.

(B) Generation of cell lines with 41-bp deletion at rs41298798 regulatory region. The MG63 cell line was edited to generate three homozygous clones for the control and bi-allelic 41-bp deletion (Δ41/Δ41), with two single-guide RNAs flanking rs41298798.

(C) MG63 cells with the Δ41 deletion showed dysregulated expression levels of genes at the 22q11.21 locus. The results were normalized to *GAPDH* levels ($n = 3$ per genotype; the results are presented as mean ± SEM; $^{**}p < 0.01$; $^{***}p < 0.001$; NS, not significant; Student's t test).

(D) Generation of edited MG63 cells at rs41298798. The MG63 cell line was edited to generate three homozygous clones for both the derived allele rs41298798-C and the ancestral allele rs41298798-G. ssODN, single-stranded oligodeoxynucleotide.

(E) The 3D Interaction Viewer (3DIV) with the hg38 genome assembly showing the genomic context of chromosome 22q11.21 (top). The rs41298798-G/G MG63 cells show reduced expression of *TBX1* compared to the C/C isogenic control (bottom). The results for each sample were normalized to its *GAPDH* content ($n = 3$ per genotype; the results are presented as mean ± SEM; $^{*}p \leq 0.05$; $^{**}p \leq 0.01$; $^{****}p \leq 0.0001$; NS, not significant; Student's t test).

inclined ventrally toward the displaced basioccipital bones, resulting in a forward-inclined neck (Figures 4B and 4C). After the premature fusion of SOS,[39] mesoderm-specific *Tbx1*-KO embryos (*Tbx1$^{loxP/KO}$;Mesp1-Cre*) exhibited phenotypes that recapitulated those of *Tbx1*-KO embryos (Figure S8A). As previously reported,[35,37,38] *Tbx1*-KO mice were deficient in the anterior arch of C1 (the atlas), lacked the hyoid bone, and had reduced and fragmented thyroid cartilage (Figure 4C). The lower vertical odontoid process of C2 and the absence of the anterior arch of C1 reduced the sagittal space of the oropharynx and hypopharynx in *Tbx1*-KO embryos (Figure 4B). In the developing vertebral column, segmental expression of *Tbx1* begins on embryonic day (E) 9.5, and *Tbx1* localizes to each sclerotome at E12.5.[66] *Tbx1*-KO mice have shortened necks.[35] Therefore, we examined the vertebral column of *Tbx1*-KO mice. Consistently, *Tbx1*-KO neonates showed hypoplasia of the cervical vertebrae (Figures 4C and 4D). The dorsal heights and widths of the ossified lesions of the cervical vertebrae were significantly reduced, and the C2 angle was flattened in *Tbx1*-KO mice (Figures 4C and 4D). In the lumbar vertebrae, the ossification centers of the vertebral bodies were also flattened in *Tbx1*-KO (Figure 4E) and osteochondroprogenitor-specific *Tbx1*-KO (*Tbx1$^{loxP/KO}$;Twist2-Cre*) neonates (Figure S8B). In the long bones of the forelimb and hindlimb, *Tbx1*-KO and

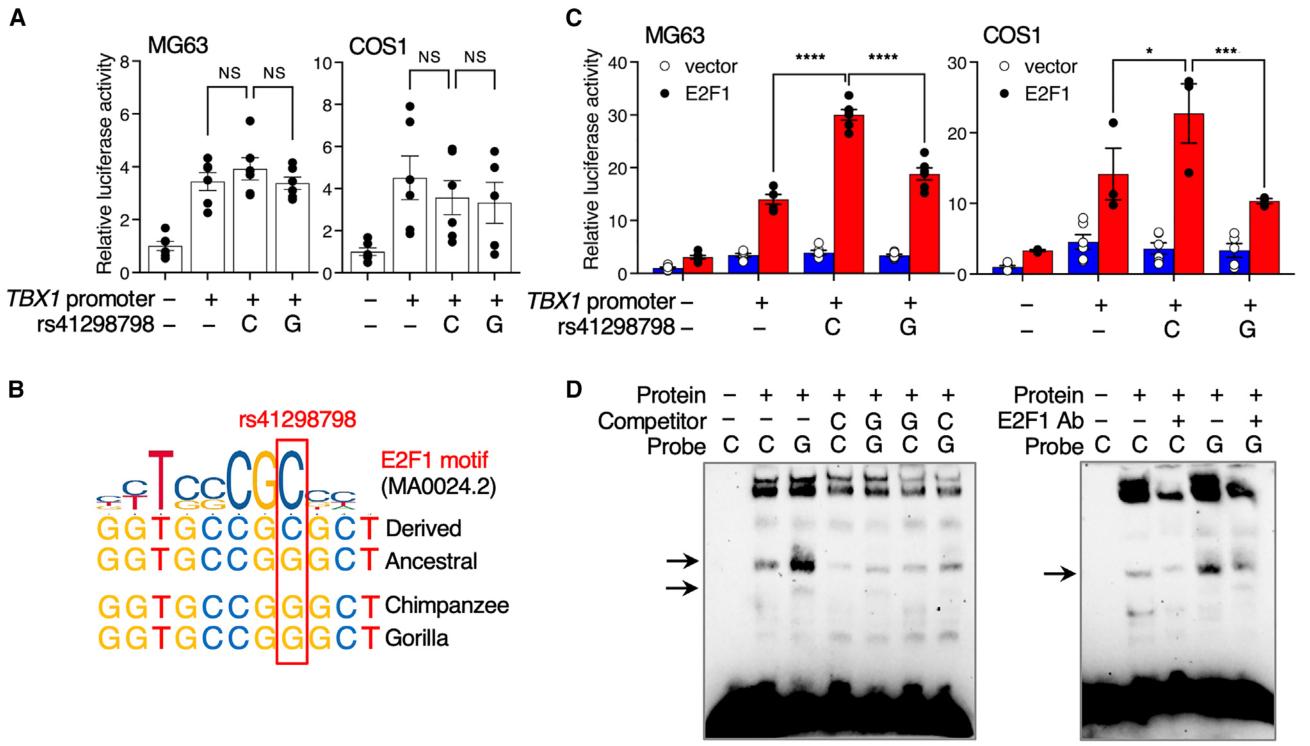

Figure 3. The ancestral allele at rs41298798 alters an E2F1-binding site and reduces E2F1 responsiveness
(A) The relative luciferase activity of constructs containing the rs41298798-C or rs41298798-G allele in MG63 and COS1 cells ($n = 6$). The results are presented as mean ± SEM; NS, not significant; one-way ANOVA.
(B) The sequence surrounding rs41298798 resembles a consensus E2F1-binding motif (JASPAR ID: MA0024.2). The derived rs41298798-C to the ancestral rs41298798-G within the predicted E2F1 binding motif. The ancestral allele is conserved in apes.
(C) Effect of *E2F1* overexpression on allele-specific enhancer activity of rs41298798 toward *TBX1*. The rs41298798-C or rs41298798-G alleles were cloned downstream of *TBX1*-promoter-driven luciferase constructs, and luciferase reporter assays were performed following transient transfection of MG63 or COS1 cells ($n = 6$; the results are presented as mean ± SEM; $^{*}p \leq 0.05$; $^{***}p \leq 0.001$; $^{****}p \leq 0.0001$; two-way analysis of variance [ANOVA]).
(D) Electrophoretic mobility shift assays (EMSAs) with biotin-labelled probes containing rs41298798-C or rs41298798-G alleles in E2F1-transfected COS1 cells. (Left) The competitor represents 200-fold excess amounts of an unlabelled probe compared with the biotin-labelled probe. (Right) EMSA using an anti-E2F1 antibody (Ab). Black arrows, allele-specific bands that interact with nuclear proteins. Uncropped images are shown in Figure S9.

Tbx1$^{loxP/KO}$;*Mesp1-Cre* neonates showed approximately a 15% reduction in the length of the ossified shaft at the ulna and tibia (Figures 4F and S8C). These results indicate that a reduced dosage of *Tbx1* leads to specific changes in the skeletal morphology of mice.

Neanderthals exhibited skeletal phenotypes reminiscent of TBX1 deficiency

TBX1 expression was downregulated by the ancestral allele compared to the derived allele (Figure 2E). The effect of rs41298798 on *TBX1* promoter activity showed a significant allelic difference when co-transfected with E2F1 (Figure 3C). Therefore, we hypothesized that phenotypes similar to those driven by *TBX1* dosage may also exist between *Homo sapiens* and extinct hominins. To investigate whether extinct hominins may have TBX1-deficient-like phenotypes, we collected information on divergent phenotypes in the skull base and vertebral column of Neanderthals (Tables 2 and S4). We tested whether each known phenotypic difference was present in *Tbx1*-KO mice (Tables 2 and S4). We found that 7 out of 7 phenotypes showed the same directionality between wild-type and *Tbx1*-KO mice as they do between modern humans and Neanderthals (100% compared with 50% expected by chance, $p = 7.8 \times 10^{-3}$, binomial test; Figure 5A). In other words, *Tbx1*-KO mouse phenotypes differ from modern human phenotypes in the skull base and vertebral column and mirror ancestral states. These results suggest that the degree of phenotypic change in the skull base and vertebrae is *TBX1*-expression dependent.

Humans with DGS/VCFS have skeletal and cranial anomalies, including a shortened posterior skull base length, platybasia, and dysmorphic vertebrae (Tables 2 and S4). While DGS/VCFS results from a *de novo* heterozygous deletion of chromosome 22q11.2 or loss-of-function mutations in the *TBX1* coding region,[26–29] an ancestral allele of rs41298798 may replicate some of the effects of *TBX1* haploinsufficiency by reducing *TBX1* expression. To investigate the association between TBX1 downregulation and the corresponding skull base and vertebral phenotypes in modern humans versus Neanderthals, we tested whether *TBX1* haploinsufficiency phenotypes resemble

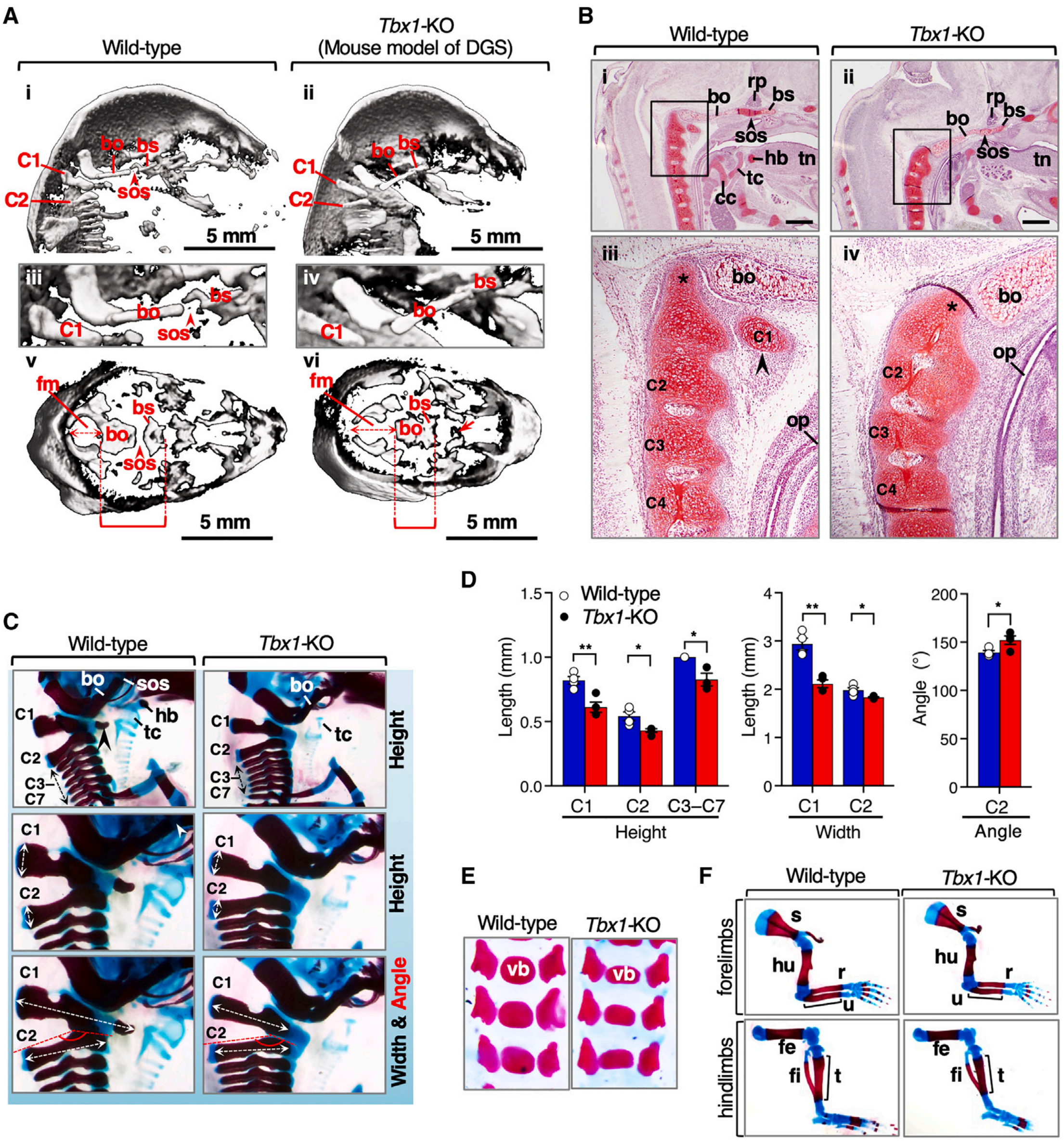

Figure 4. Key skeletal phenotypes observed in *Tbx1*-KO mice

(A) Skulls from wild-type and *Tbx1*-KO neonates were subjected to micro-computed tomography and are shown as one sagittal plane through the skull base (i–iv) at low (i and ii) and high (iii and iv) magnification and by bird's-eye view (v and vi). The spheno-occipital synchondrosis (SOS) is depicted as the space between the basisphenoid (bs) and basioccipital (bo) bones in wild-type mice (i, iii, and v). Note that the fusion of adjacent bones (bs and bo) in *Tbx1*-KO mice (vi) reduces the anteroposterior length of the posterior region of the skull base (red bracket) and elongates the foramen magnum (fm) anteroposteriorly. *Tbx1*-KO mice have a cleft palate[65] (red arrow in vi).

(B) Sagittal sections of E14.5 wild-type (i and iii) and *Tbx1*-KO (ii and iv) embryos were stained with safranin O/haematoxylin/Fast Green and observed at low (i and ii) and high (iii and iv) magnification. Ar, the anterior arch of C1. Note that the C2 odontoid process (asterisk) is tilted ventrally toward the malformed skull base in *Tbx1*-KO embryos (iv). Abnormal intraoral epithelial adhesion is observed between the posterior domain of the *Tbx1*-KO palate and the oropharynx[65] (ii). rp, Rathke's pouch; hb, hyoid bone; tc, thyroid cartilage; cc, cricoid cartilage; tn, tongue; op, oropharynx. Scale bars: 0.4 mm.

(C) Alizarin red and Alcian blue staining of bones of the lateral view of the cervical vertebrae (C1–C7) of wild-type and *Tbx1*-KO mice. In *Tbx1*-KO neonates, the cervical vertebrae are hypoplastic, the anterior arch of C1 (ar) is missing, the body of the hyoid bone (hb) is absent, and the thyroid cartilages (tc) are hypoplastic. Diagrams of the vertebrae with the landmarks for the measured parameters in (D) are also shown.

(D) Measurements of vertebrae from wild-type and *Tbx1*-KO mice ($n = 4$ for each genotype; the results are presented as mean ± SEM; *$p < 0.05$; **$p < 0.01$; Student's t test).

(E) Ventral view of lumbar vertebrae (L1–L3) from wild-type and *Tbx1*-KO neonates. vb, vertebral body.

(F) Staining of bones of the forelimbs and hindlimbs of wild-type and *Tbx1*-KO neonates. Brackets indicate the ossified shaft of the ulna (u) and tibia (t). s, scapula; hu, humerus; r, radius; fe, femur; fi, fibula.

Table 2. The skull base and vertebral phenotypes in Neanderthals and individuals with DGS/VCFS compared to modern humans, and the phenotypes that differ between *Tbx1*-KO and wild-type mice

		Humans			Newborn mice	
		**Modern**	**DGS/VCFS**	**Neanderthals**	***Tbx1*-KO**	**Wild-type**
TBX1, Tbx1	gene dosage	100%	50%	N/A	0%	100%
	gene expression	100%	predicted to be lower	predicted to be lower	0%	100%
**Skull base and vertebral morphology**						
Skull base	platybasia	control	+	+	+ (this study)	control
	shorter length of posterior skull base	control	+	+	+	control
	elongated foramen magnum	control	+ (this study)	+	+ (this study)	control
	basilar impression	control	+	N/A	N/A	control
Vertebral column	hypoplastic or anomalous atlas (C1)	control	+	+	+	control
	dysmorphic axis (C2)	control	+	+	+	control
	fusion of C1–C2	control	+	+	N/A	control
	fusion of C2–C3	control	+	+	N/A	control
	lower dorsal height of the C2–C7	control	+	+	+ (this study)	control
	platyspondyly (cervical and thoracic)	control	+	+	+ (this study)	control
**Other traits**						
Limb	distal shortening of limbs	control	N/A	+	+ (this study)	control
Neck	short neck	control	+	+	+	control
Height	short stature	control	+	+	+	control

"+" represents the present phenotype. N/A, not available. Full details are presented in Table S4.

phenotypes that differ among hominins. Individuals with DGS/VCFS exhibit a shortened skull base.[34] We compared the cephalometric data of individuals with DGS/VCFS and controls, focusing on the longitudinal diameter of the foramen magnum (Figure S10). The average longitudinal diameter was 18.3 ± 7.7 mm in the controls and 23.3 ± 7.0 mm in the individuals with DGS/VCFS ($n = 41$ for each group; mean ± standard deviation; paired Student's t test, $p < 0.0001$). This result indicates that the foramen magnum is anteroposteriorly elongated in individuals with DGS/VCFS (Table 2). We found that 9 out of 9 DGS/VCFS phenotypes (100%) match the directionality of the evolutionary contrasts discussed above, compared to 50% expected by chance ($p = 2.0 \times 10^{-3}$, binomial test; Figure 5B). Notably, the key phenotypes that are often used to describe differences in skeletal phenotypes between modern humans and Neanderthals were observed in individuals with DGS/VCFS as well as in *Tbx1*-KO mice (Tables 2 and S4). These results suggest that the skeletal phenotypic manifestation is *TBX1*-expression dependent and is consistent between Neanderthals and individuals with *TBX1* haploinsufficiency where *TBX1* expression is predicted to be lower than in healthy humans. As depicted in our model (Figure 5C), our findings suggest the association between the functional SNP rs41298798 and the basicranial morphology of humans. Moreover, our results suggest that the mechanism by which this SNP controls *TBX1* expression may have contributed to the evolution of the human skull base and vertebral column.

Discussion

The majority of Neanderthal alleles are not highly adaptive, resulting in low frequencies (<2%) in modern humans.[67] To dissect ancestral alleles, the identification of the target genes of actual causal variants and their potential network is critical. Here, we showed that a regulatory component located in an intron of *TBX1* and encompassing rs41298798 affects the expression of multiple genes associated with 22q11.2 deletion syndrome, including *TBX1*. Of note, the ancestral allele of rs41298798 drives a decrease in *TBX1* expression levels of approximately 59% compared to the expression levels of the derived allele. The effect of the ancestral allele may have been significant because *TBX1* haploinsufficiency induces DGS/VCFS.[26–29] We demonstrated that an intronic SNP, rs41298798, acts as an allele-specific enhancer to induce *TBX1* transcriptional activity mediated by E2F1. E2F1 binding at rs41298798 increases transcriptional activation of the *TBX1* promoter, revealing that the derived rs41298798-C allele has higher transcriptional activity than the ancestral rs41298798-G allele. E2F1 is a critical TF that recruits the RNA polymerase II cofactor to mediate enhancer-promoter interactions that affect gene

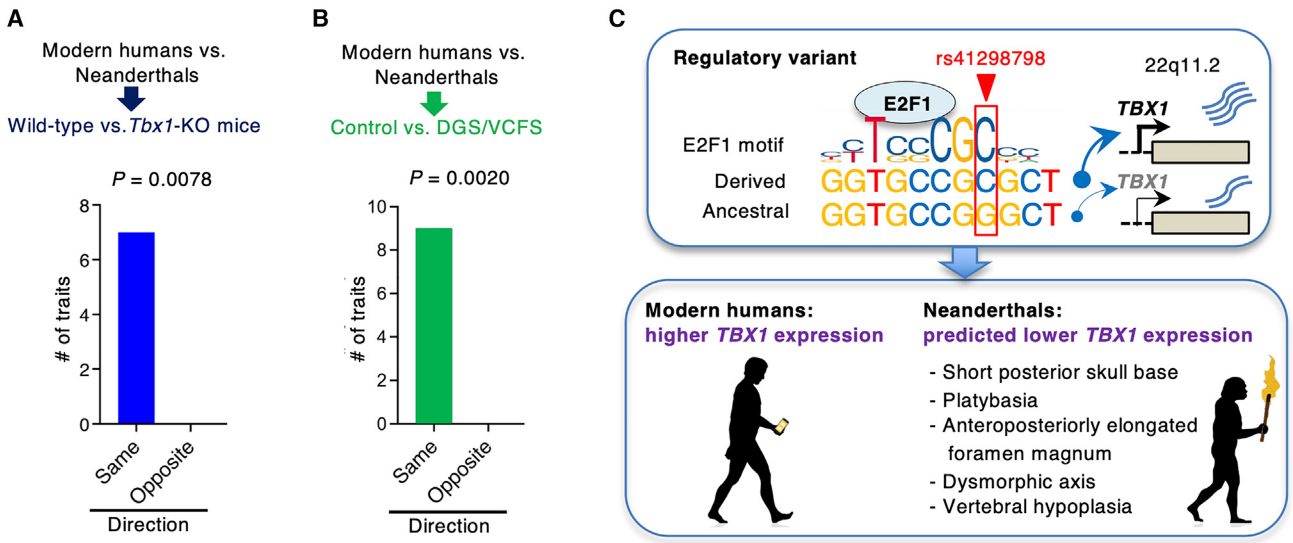

Figure 5. Phenotypic differences associated with *TBX1* deficiency are observed between modern humans (*Homo sapiens*) and Neanderthals (*Homo neanderthalensis*)
(A) The number of the skull base and vertebral phenotypes that are similar between modern humans and Neanderthals and between wild-type and *Tbx1*-KO mice (Table 2). Two-sided binomial test *p* values are shown. Phenotypic differences in wild-type versus *Tbx1*-KO mice mirror the phenotypic differences in modern humans versus Neanderthals.
(B) The number of identical skull base and vertebral phenotypes between modern humans and Neanderthals and between healthy and DGS/VCFS individuals (Table 2). Two-sided binomial test *p* values are shown. Phenotypic differences between healthy individuals and those with DGS/VCFS mirror the phenotypic differences between modern humans and Neanderthals.
(C) Summary of the enhancer activity of rs41298798 with a proposed model for the basicranial morphology found in *Homo sapiens*. A model of the rs41298798-dependent expression of *TBX1* in a coordinator motif bound with E2F1.

expression.[68,69] Thus, E2F1 may act as a mediator of allele-specific enhancer activity through rs41298798, thereby strengthening enhancer-promoter interactions and controlling *TBX1* expression. In addition to its effect on *TBX1*, the allelic variation at rs41298798 induces the dysregulation of genes located within the 22q11.21 locus. These genes may collectively exert a synergistic influence on the phenotype. Further studies are needed to determine how the regulatory elements in *TBX1* introns mediate gene expression at 22q11.21. Interestingly, the ancestral alleles in the *TBX1* locus are more prevalent in South Asians and Japanese, which is consistent with previous reports indicating that Neanderthals share more ancestral alleles with East Asians than with Europeans.[70–72] These findings may reflect additional interbreeding in the ancestors of East Asians.[73]

It has long been unclear whether the various cranial features of modern humans have evolved in response to separate selective pressures or whether they are the result of inherent morphological integration of the skull.[21] To investigate whether changes in *TBX1* expression contribute to the morphological changes in basicranial morphology, we compared skeletal phenotypes, focusing on the skull base and vertebrae. *Tbx1*-KO mice show skull base phenotypes similar to the divergent phenotypes of modern humans and Neanderthals. Individuals with DGS/VCFS also exhibit phenotypes that can help infer the effect of ancestral alleles. Thus, *TBX1* haploinsufficiency phenotypes suggest that *TBX1* upregulation may have been involved in morphological changes in the skull base during human evolution. The increased length of the skull base is mainly driven by SOS.[22] Precocious ossification and/or malformation of the SOS causes the fusion of the basisphenoid and basioccipital bones and subsequent malformations leading to a shortened posterior region of the skull base and platybasia, which in turn causes an anteroposteriorly elongated foramen magnum and cervical malposition. In other words, the *TBX1* dosage affects the length, morphology, and angle of the skull base and induces subsequent changes in the C2 odontoid process. *Tbx1*-KO mice are deficient in the anterior arch of C1 and exhibit hypomorphic vertebrae. These phenotypes may play a role in the loss of the forwardly inclined neck in the *Homo sapiens* lineage, because species with vertically oriented odontoid processes can position the head perpendicular to the neck, allowing the weight of the head to be better supported by the vertebral column.[74] They may also go hand-in-hand with modified brain ontogeny, allowing specific brain regions to expand.[75] The study of cartilage in extinct *Homo* lineages remains challenging[76]; however, our results raise the possibility that *TBX1* upregulation in *Homo sapiens* may be associated with changes in ancestral traits and/or disease susceptibilities. It is important to note that the clinical features of DGS/VCFS are highly variable, even among individuals with identical deletions,[77] suggesting that genetic background, unusual modes of inheritance, and/or environmental risk factors may affect the presentation of the phenotype.

In conclusion, our research suggests that regulatory divergence within the *TBX1* locus plays an essential role in shaping the distinctive posterior skull base and vertebral structures found in *Homo sapiens*. Further identification of causal variants, coupled with the exploration of their target gene networks, may provide insights into the evolutionary mechanisms responsible for the characteristic morphology of *Homo sapiens*.

Data and code availability

The cephalometric data from individuals with DGS/VCFS was collected in a clinical setting. Individual-level cephalometric measurements are not available due to current data protection legislation. The published article includes all other datasets generated or analyzed during this study.

Supplemental information

Supplemental information can be found online at https://doi.org/10.1016/j.ajhg.2024.03.012.

Acknowledgments

We thank Deepak Srivastava, David Ornitz, Michelle Tallquist, and Yumiko Saga for providing the mice; Feng Zhang and Masataka Nakamura for providing the reagents; Hiromi Yanagisawa, David Bick, and Juan Moriano for discussions; and Eriko Matsumoto for technical assistance. The cell lines were provided by RIKEN BRC through the National BioResource Project of MEXT Japan or the Cell Resource Center for Biomedical Research, Tohoku University. This work was supported by grants from the Japan Society for the Promotion of Science (JSPS) (KAKENHI grant numbers JP20K09901 and JP23K09149) (N.F.), and the Spanish Ministry of Science and Innovation (PID2019-107042GB-I00) (C.B.).

Author contributions

N.F. provided overall project management. N.F. and C.B. discussed and evaluated the *in silico* data. N.F. designed and performed all the *in vitro* and *in vivo* experiments. A.H. collected and managed the cephalometric samples and clinical data. All authors discussed the results and implications and commented on the manuscript at all stages.

Declaration of interests

The authors declare no competing interests.

Received: October 30, 2023
Accepted: March 15, 2024
Published: April 11, 2024

Web resources

1000 Genomes Project, http://www.1000genomes.org/
3DIV & database, http://kobic.kr/3div/
3DSNP, http://cbportal.org/3dsnp
CADD, https://cadd.gs.washington.edu/
CHOPCHOP, https://chopchop.cbu.uib.no/
dbSNP, http://www.ncbi.nlm.nih.gov/snp/
ENCODE, https://www.encodeproject.org/
Ensembl, http://asia.ensembl.org/Homo_sapiens/Info/Index
GEPIA, http://gepia.cancer-pku.cn/detail.php
GTEx project, http://www.gtexportal.org/home
HaploReg, https://pubs.broadinstitute.org/mammals/haploreg/haploreg.php
JASPAR 2020 CORE, http://jaspar.elixir.no
MGI, https://www.informatics.jax.org/
Monarch Initiative, https://monarchinitiative.org
NCBI Gene, https://www.ncbi.nlm.nih.gov/gene/
OMIM, http://www.omim.org/
PubMed, https://pubmed.ncbi.nlm.nih.gov/
RegulomeDB, http://regulomedb.org
TCGA, https://tcga-data.nci.nih.gov/
UCSC Genome Browser, https://genome.ucsc.edu/

References

1. King, M.C., and Wilson, A.C. (1975). Evolution at two levels in humans and chimpanzees. Science *188*, 107–116. https://doi.org/10.1126/science.1090005.
2. Balzeau, A., Albessard-Ball, L., Kubicka, A.M., Filippo, A., Beaudet, A., Santos, E., Bienvenu, T., Arsuaga, J.-L., Bartsiokas, A., Berger, L., et al. (2022). Dosage analysis of the 7q11.23 Williams region identifies BAZ1B as a major human gene patterning the modern human face and underlying self-domestication Matteo. Sci. Adv. *8*, eabp9767. https://doi.org/10.1126/sciadv.abp9767.
3. Kuhlwilm, M., and Boeckx, C. (2019). A catalog of single nucleotide changes distinguishing modern humans from archaic hominins. Sci. Rep. *9*, 8463. https://doi.org/10.1038/s41598-019-44877-x.
4. Peyrégne, S., Boyle, M.J., Dannemann, M., and Prüfer, K. (2017). Detecting ancient positive selection in humans using extended lineage sorting. Genome Res. *27*, 1563–1572. https://doi.org/10.1101/gr.219493.116.
5. Weyer, S., and Pääbo, S. (2016). Functional analyses of transcription factor binding sites that differ between present-day and archaic humans. Mol. Biol. Evol. *33*, 316–322. https://doi.org/10.1093/molbev/msv215.
6. Franke, M., Ibrahim, D.M., Andrey, G., Schwarzer, W., Heinrich, V., Schöpflin, R., Kraft, K., Kempfer, R., Jerković, I., Chan, W.L., et al. (2016). Formation of new chromatin domains determines pathogenicity of genomic duplications. Nature *538*, 265–269. https://doi.org/10.1038/nature19800.
7. Long, H.K., Prescott, S.L., and Wysocka, J. (2016). Ever-changing landscapes: Transcriptional enhancers in development and evolution. Cell *167*, 1170–1187. https://doi.org/10.1016/j.cell.2016.09.018.
8. Wang, X., Zhou, T., Wunderlich, Z., Maurano, M.T., DePace, A.H., Nuzhdin, S.V., and Rohs, R. (2018). Analysis of genetic variation indicates DNA shape involvement in purifying selection. Mol. Biol. Evol. *35*, 1958–1967. https://doi.org/10.1093/molbev/msy099.
9. Gokhman, D., Agoglia, R.M., Kinnebrew, M., Gordon, W., Sun, D., Bajpai, V.K., Naqvi, S., Chen, C., Chan, A., Chen, C., et al. (2021). Human-chimpanzee fused cells reveal cis-regulatory

divergence underlying skeletal evolution. Nat. Genet. *53*, 467–476. https://doi.org/10.1038/s41588-021-00804-3.

10. Silvert, M., Quintana-Murci, L., and Rotival, M. (2019). Impact and evolutionary determinants of Neanderthal introgression on transcriptional and post-transcriptional regulation. Am. J. Hum. Genet. *104*, 1241–1250. https://doi.org/10.1016/j.ajhg.2019.04.016.
11. Chen, X.F., Zhu, D.L., Yang, M., Hu, W.X., Duan, Y.Y., Lu, B.J., Rong, Y., Dong, S.S., Hao, R.H., Chen, J.B., et al. (2018). An osteoporosis risk SNP at 1p36.12 acts as an allele-specific enhancer to modulate LINC00339 expression via long-range loop formation. Am. J. Hum. Genet. *102*, 776–793. https://doi.org/10.1016/j.ajhg.2018.03.001.
12. Mika, K.M., Li, X., DeMayo, F.J., and Lynch, V.J. (2018). An ancient fecundability-associated polymorphism creates a GATA2 binding site in a distal enhancer of HLA-F. Am. J. Hum. Genet. *103*, 509–521. https://doi.org/10.1016/j.ajhg.2018.08.009.
13. Gazal, S., Weissbrod, O., Hormozdiari, F., Dey, K.K., Nasser, J., Jagadeesh, K.A., Weiner, D.J., Shi, H., Fulco, C.P., O'Connor, L.J., et al. (2022). Combining SNP-to-gene linking strategies to identify disease genes and assess disease omnigenicity. Nat. Genet. *54*, 827–836. https://doi.org/10.1038/s41588-022-01087-y.
14. Shendure, J., and Akey, J.M. (2015). The origins, determinants, and consequences of human mutations. Science *349*, 1478–1483. https://doi.org/10.1126/science.aaa9119.
15. Pääbo, S. (2014). The human condition-a molecular approach. Cell *157*, 216–226. https://doi.org/10.1016/j.cell.2013.12.036.
16. Gokhman, D., Mishol, N., de Manuel, M., de Juan, D., Shuqrun, J., Meshorer, E., Marques-Bonet, T., Rak, Y., and Carmel, L. (2019). Reconstructing Denisovan anatomy using DNA methylation maps. Cell *179*, 180–192.e10. https://doi.org/10.1016/j.cell.2019.08.035.
17. Neubauer, S., Hublin, J.-J., and Gunz, P. (2018). The evolution of modern human brain shape. Sci. Adv. *4*, eaao5961. https://doi.org/10.1126/sciadv.aao5961.
18. Lesciotto, K.M., and Richtsmeier, J.T. (2019). Craniofacial skeletal response to encephalization: How do we know what we think we know? Am. J. Phys. Anthropol. *168* (*Suppl*), 27–46. https://doi.org/10.1002/ajpa.23766.
19. Lieberman, D.E., and McCarthy, R.C. (1999). The ontogeny of cranial base angulation in humans and chimpanzees and its implications for reconstructing pharyngeal dimensions. J. Hum. Evol. *36*, 487–517. https://doi.org/10.1006/jhev.1998.0287.
20. Weaver, T.D. (2009). Out of Africa: modern human origins special feature: the meaning of neandertal skeletal morphology. Proc. Natl. Acad. Sci. USA *106*, 16028–16033. https://doi.org/10.1073/pnas.0903864106.
21. Martínez-Abadías, N., Esparza, M., Sjøvold, T., González-José, R., Santos, M., Hernández, M., and Klingenberg, C.P. (2012). Pervasive genetic integration directs the evolution of human skull shape. Evolution *66*, 1010–1023. https://doi.org/10.1111/j.1558-5646.2011.01496.x.
22. Madeline, L.A., and Elster, A.D. (1995). Postnatal development of the central skull base: normal variants. Radiology *196*, 757–763. https://doi.org/10.1148/radiology.196.3.7644640.
23. Funato, N. (2020). New insights into cranial synchondrosis development: A mini review. Front. Cell Dev. Biol. *8*, 706. https://doi.org/10.3389/fcell.2020.00706.
24. Haeusler, M., Trinkaus, E., Fornai, C., Müller, J., Bonneau, N., Boeni, T., and Frater, N. (2019). Morphology, pathology, and the vertebral posture of the La Chapelle-aux-Saints Neandertal. Proc. Natl. Acad. Sci. USA *116*, 4923–4927. https://doi.org/10.1073/pnas.1820745116.
25. Schiess, R., Boeni, T., Rühli, F., and Haeusler, M. (2014). Revisiting scoliosis in the KNM-WT 15000 Homo erectus skeleton. J. Hum. Evol. *67*, 48–59. https://doi.org/10.1016/j.jhevol.2013.12.009.
26. Yagi, H., Furutani, Y., Hamada, H., Sasaki, T., Asakawa, S., Minoshima, S., Ichida, F., Joo, K., Kimura, M., Imamura, S.i., et al. (2003). Role of TBX1 in human del22q11.2 syndrome. Lancet (London, England) *362*, 1366–1373.
27. Zweier, C., Sticht, H., Aydin-Yaylagül, I., Campbell, C.E., and Rauch, A. (2007). Human TBX1 missense mutations cause gain of function resulting in the same phenotype as 22q11.2 deletions. Am. J. Hum. Genet. *80*, 510–517. https://doi.org/10.1086/511993.
28. Jaouadi, A., Tabebi, M., Abdelhedi, F., Abid, D., Kamoun, F., Chabchoub, I., Maatoug, S., Doukali, H., Belghuith, N., Ksentini, M.A., et al. (2018). A novel TBX1 missense mutation in patients with syndromic congenital heart defects. Biochem. Biophys. Res. Commun. *499*, 563–569. https://doi.org/10.1016/j.bbrc.2018.03.190.
29. Paylor, R., Glaser, B., Mupo, A., Ataliotis, P., Spencer, C., Sobotka, A., Sparks, C., Choi, C.H., Oghalai, J., Curran, S., et al. (2006). Tbx1 haploinsufficiency is linked to behavioral disorders in mice and humans: Implications for 22q11 deletion syndrome. Proc. Natl. Acad. Sci. USA *103*, 7729–7734. https://doi.org/10.1073/pnas.0600206103.
30. Bassett, A.S., Chow, E.W.C., Husted, J., Weksberg, R., Caluseriu, O., Webb, G.D., and Gatzoulis, M.A. (2005). Clinical features of 78 adults with 22q11 deletion syndrome. Am. J. Med. Genet. *138*, 307–313. https://doi.org/10.1002/ajmg.a.30984.
31. Ming, J.E., McDonald-McGinn, D.M., Megerian, T.E., Driscoll, D.A., Elias, E.R., Russell, B.M., Irons, M., Emanuel, B.S., Markowitz, R.I., and Zackai, E.H. (1997). Skeletal anomalies and deformities in patients with deletions of 22q11. Am. J. Med. Genet. *72*, 210–215. https://doi.org/10.1002/(sici)1096-8628(19971017)72:2<210::aid-ajmg16>3.0.co;2-q.
32. Ricchetti, E.T., States, L., Hosalkar, H.S., Tamai, J., Maisenbacher, M., McDonald-McGinn, D.M., Zackai, E.H., and Drummond, D.S. (2004). Radiographic study of the upper cervical spine in the 22Q11.2 deletion syndrome. J. Bone Jt. Surg. *86*, 1751–1760. https://doi.org/10.2106/00004623-200408000-00020.
33. McDonald-McGinn, D.M., Kirschner, R., Goldmuntz, E., Sullivan, K., Eicher, P., Gerdes, M., Moss, E., Solot, C., Wang, P., Jacobs, I., et al. (1999). The Philadelphia story: the 22q11.2 deletion: report on 250 patients. Genet. Couns. *10*, 11–24.
34. Heliövaara, A., and Hurmerinta, K. (2006). Craniofacial cephalometric morphology in children with CATCH 22 syndrome. Orthod. Craniofac. Res. *9*, 186–192. https://doi.org/10.1111/j.1601-6343.2006.00373.x.
35. Jerome, L.A., and Papaioannou, V.E. (2001). DiGeorge syndrome phenotype in mice mutant for the T-box gene, Tbx1. Nat. Genet. *27*, 286–291. https://doi.org/10.1038/85845.
36. Lindsay, E.A., Vitelli, F., Su, H., Morishima, M., Huynh, T., Pramparo, T., Jurecic, V., Ogunrinu, G., Sutherland, H.F., Scambler, P.J., et al. (2001). Tbx1 haploinsufficieny in the DiGeorge syndrome region causes aortic arch defects in mice. Nature *410*, 97–101. https://doi.org/10.1038/35065105.

37. Hu, T., Yamagishi, H., Maeda, J., McAnally, J., Yamagishi, C., and Srivastava, D. (2004). Tbx1 regulates fibroblast growth factors in the anterior heart field through reinforcing autoregulatory loop involving forkhead transcription factors. Development *131*, 5491–5502. https://doi.org/10.1242/dev.01399.
38. Funato, N., Nakamura, M., Richardson, J.A., Srivastava, D., and Yanagisawa, H. (2015). Loss of Tbx1 induces bone phenotypes similar to cleidocranial dysplasia. Hum. Mol. Genet. *24*, 424–435. https://doi.org/10.1093/hmg/ddu458.
39. Funato, N., Srivastava, D., Shibata, S., and Yanagisawa, H. (2020). TBX1 regulates chondrocyte maturation in the spheno-occipital synchondrosis. J. Dent. Res. *99*, 1182–1191. https://doi.org/10.1177/0022034520925080.
40. 1000 Genomes Project Consortium, Auton, A., Brooks, L.D., Durbin, R.M., Garrison, E.P., Kang, H.M., Korbel, J.O., Marchini, J.L., McCarthy, S., McVean, G.A., and Abecasis, G.R. (2015). A global reference for human genetic variation. Nature *526*, 68–74. https://doi.org/10.1038/nature15393.
41. Rentzsch, P., Witten, D., Cooper, G.M., Shendure, J., and Kircher, M. (2019). CADD: predicting the deleteriousness of variants throughout the human genome. Nucleic Acids Res. *47*, D886–D894. https://doi.org/10.1093/nar/gky1016.
42. Ward, L.D., and Kellis, M. (2016). HaploReg v4: systematic mining of putative causal variants, cell types, regulators and target genes for human complex traits and disease. Nucleic Acids Res. *44*, D877–D881. https://doi.org/10.1093/nar/gkv1340.
43. Quan, C., Ping, J., Lu, H., Zhou, G., and Lu, Y. (2022). 3DSNP 2.0: update and expansion of the noncoding genomic variant annotation database. Nucleic Acids Res. *50*, D950–D955. https://doi.org/10.1093/nar/gkab1008.
44. Boyle, A.P., Hong, E.L., Hariharan, M., Cheng, Y., Schaub, M.A., Kasowski, M., Karczewski, K.J., Park, J., Hitz, B.C., Weng, S., et al. (2012). Annotation of functional variation in personal genomes using RegulomeDB. Genome Res. *22*, 1790–1797. https://doi.org/10.1101/gr.137323.112.
45. ENCODE Project Consortium, Moore, J.E., Purcaro, M.J., Pratt, H.E., Epstein, C.B., Shoresh, N., Adrian, J., Kawli, T., Davis, C.A., Dobin, A., et al. (2020). Expanded encyclopaedias of DNA elements in the human and mouse genomes. Nature *583*, 699–710. https://doi.org/10.1038/s41586-020-2493-4.
46. GTEx Consortium (2015). Human genomics. The Genotype-Tissue Expression (GTEx) pilot analysis: multitissue gene regulation in humans. Science *348*, 648–660. https://doi.org/10.1126/science.1262110.
47. Dixon, J.R., Jung, I., Selvaraj, S., Shen, Y., Antosiewicz-Bourget, J.E., Lee, A.Y., Ye, Z., Kim, A., Rajagopal, N., Xie, W., et al. (2015). Chromatin architecture reorganization during stem cell differentiation. Nature *518*, 331–336. https://doi.org/10.1038/nature14222.
48. Yang, D., Jang, I., Choi, J., Kim, M.S., Lee, A.J., Kim, H., Eom, J., Kim, D., Jung, I., and Lee, B. (2018). 3DIV: A 3D-genome Interaction Viewer and database. Nucleic Acids Res. *46*, D52–D57. https://doi.org/10.1093/nar/gkx1017.
49. Tang, Z., Li, C., Kang, B., Gao, G., Li, C., and Zhang, Z. (2017). GEPIA: a web server for cancer and normal gene expression profiling and interactive analyses. Nucleic Acids Res. *45*, W98–W102. https://doi.org/10.1093/nar/gkx247.
50. Cancer Genome Atlas Research Network, Weinstein, J.N., Collisson, E.A., Mills, G.B., Shaw, K.R.M., Ozenberger, B.A., Ellrott, K., Shmulevich, I., Sander, C., and Stuart, J.M. (2013). The Cancer Genome Atlas Pan-Cancer analysis project. Nat. Genet. *45*, 1113–1120. https://doi.org/10.1038/ng.2764.
51. Fornes, O., Castro-Mondragon, J.A., Khan, A., van der Lee, R., Zhang, X., Richmond, P.A., Modi, B.P., Correard, S., Gheorghe, M., Baranašić, D., et al. (2020). JASPAR 2020: update of the open-access database of transcription factor binding profiles. Nucleic Acids Res. *48*, D87–D92. https://doi.org/10.1093/nar/gkz1001.
52. Labun, K., Montague, T.G., Krause, M., Torres Cleuren, Y.N., Tjeldnes, H., and Valen, E. (2019). CHOPCHOP v3: expanding the CRISPR web toolbox beyond genome editing. Nucleic Acids Res. *47*, W171–W174. https://doi.org/10.1093/nar/gkz365.
53. Ran, F.A., Hsu, P.D., Wright, J., Agarwala, V., Scott, D.A., and Zhang, F. (2013). Genome engineering using the CRISPR-Cas9 system. Nat. Protoc. *8*, 2281–2308. https://doi.org/10.1038/nprot.2013.143.
54. Schmittgen, T.D., Zakrajsek, B.A., Mills, A.G., Gorn, V., Singer, M.J., and Reed, M.W. (2000). Quantitative reverse transcription-polymerase chain reaction to study mRNA decay: comparison of endpoint and real-time methods. Anal. Biochem. *285*, 194–204. https://doi.org/10.1006/abio.2000.4753.
55. Johnson, D.G., Schwarz, J.K., Cress, W.D., and Nevins, J.R. (1993). Expression of transcription factor E2F1 induces quiescent cells to enter S phase. Nature *365*, 349–352. https://doi.org/10.1038/365349a0.
56. Saga, Y., Miyagawa-Tomita, S., Takagi, A., Kitajima, S., Miyazaki, J. i, and Inoue, T. (1999). MesP1 is expressed in the heart precursor cells and required for the formation of a single heart tube. Development *126*, 3437–3447.
57. Šošić, D., Richardson, J.A., Yu, K., Ornitz, D.M., and Olson, E.N. (2003). Twist regulates cytokine gene expression through a negative feedback loop that represses NF-kappaB activity. Cell *112*, 169–180. https://doi.org/10.1016/s0092-8674(03)00002-3.
58. Tallquist, M.D., and Soriano, P. (2000). Epiblast-restricted Cre expression in MORE mice: a tool to distinguish embryonic vs. extra-embryonic gene function. Genesis *26*, 113–115. https://doi.org/10.1002/(sici)1526-968x(200002)26:2<113::aid-gene3>3.0.co;2-2.
59. Caparrós-Martín, J.A., Valencia, M., Reytor, E., Pacheco, M., Fernandez, M., Perez-Aytes, A., Gean, E., Lapunzina, P., Peters, H., Goodship, J.A., and Ruiz-Perez, V.L. (2013). The ciliary Evc/Evc2 complex interacts with Smo and controls Hedgehog pathway activity in chondrocytes by regulating Sufu/Gli3 dissociation and Gli3 trafficking in primary cilia. Hum. Mol. Genet. *22*, 124–139. https://doi.org/10.1093/hmg/dds409.
60. Kheradpour, P., and Kellis, M. (2014). Systematic discovery and characterization of regulatory motifs in ENCODE TF binding experiments. Nucleic Acids Res. *42*, 2976–2987. https://doi.org/10.1093/nar/gkt1249.
61. Dunham, I., Kundaje, A., Aldred, S.F., Collins, P.J., Davis, C.A., Doyle, F., Epstein, C.B., Frietze, S., Harrow, J., Kaul, R., et al. (2012). An integrated encyclopedia of DNA elements in the human genome. Nature *489*, 57–74. https://doi.org/10.1038/nature11247.
62. Andirkó, A., Moriano, J., Vitriolo, A., Kuhlwilm, M., Testa, G., and Boeckx, C. (2022). Temporal mapping of derived high-frequency gene variants supports the mosaic nature of the evolution of Homo sapiens. Sci. Rep. *12*, 9937. https://doi.org/10.1038/s41598-022-13589-0.

63. Kou, I., Otomo, N., Takeda, K., Momozawa, Y., Lu, H.-F., Kubo, M., Kamatani, Y., Ogura, Y., Takahashi, Y., Nakajima, M., et al. (2019). Genome-wide association study identifies 14 previously unreported susceptibility loci for adolescent idiopathic scoliosis in Japanese. Nat. Commun. *10*, 3685. https://doi.org/10.1038/s41467-019-11596-w.

64. Zhao, Y., Diacou, A., Johnston, H.R., Musfee, F.I., McDonald-McGinn, D.M., McGinn, D., Crowley, T.B., Repetto, G.M., Swillen, A., Breckpot, J., et al. (2020). Complete Sequence of the 22q11.2 Allele in 1,053 Subjects with 22q11.2 Deletion Syndrome Reveals Modifiers of Conotruncal Heart Defects. Am. J. Hum. Genet. *106*, 26–40. https://doi.org/10.1016/j.ajhg.2019.11.010.

65. Funato, N., Nakamura, M., Richardson, J.A., Srivastava, D., and Yanagisawa, H. (2012). Tbx1 regulates oral epithelial adhesion and palatal development. Hum. Mol. Genet. *21*, 2524–2537. https://doi.org/10.1093/hmg/dds071.

66. Chapman, D.L., Garvey, N., Hancock, S., Alexiou, M., Agulnik, S.I., Gibson-Brown, J.J., Cebra-Thomas, J., Bollag, R.J., Silver, L.M., and Papaioannou, V.E. (1996). Expression of the T-box family genes, Tbx1-Tbx5, during early mouse development. Dev. Dyn. *206*, 379–390. https://doi.org/10.1002/(SICI)1097-0177(199608)206:4<379::AID-AJA4>3.0.CO;2-F.

67. Dannemann, M., and Kelso, J. (2017). The contribution of Neanderthals to phenotypic variation in modern humans. Am. J. Hum. Genet. *101*, 578–589. https://doi.org/10.1016/j.ajhg.2017.09.010.

68. Bidon, B., Iltis, I., Semer, M., Nagy, Z., Larnicol, A., Cribier, A., Benkirane, M., Coin, F., Egly, J.-M., and Le May, N. (2018). XPC is an RNA polymerase II cofactor recruiting ATAC to promoters by interacting with E2F1. Nat. Commun. *9*, 2610. https://doi.org/10.1038/s41467-018-05010-0.

69. Rodriguez-Bravo, V., Pippa, R., Song, W.M., Carceles-Cordon, M., Dominguez-Andres, A., Fujiwara, N., Woo, J., Koh, A.P., Ertel, A., Lokareddy, R.K., et al. (2018). Nuclear pores promote lethal prostate cancer by increasing POM121-driven E2F1, MYC, and AR nuclear import. Cell *174*, 1200–1215.e20. https://doi.org/10.1016/j.cell.2018.07.015.

70. Wall, J.D., Yang, M.A., Jay, F., Kim, S.K., Durand, E.Y., Stevison, L.S., Gignoux, C., Woerner, A., Hammer, M.F., and Slatkin, M. (2013). Higher levels of neanderthal ancestry in East Asians than in Europeans. Genetics *194*, 199–209. https://doi.org/10.1534/genetics.112.148213.

71. Vernot, B., and Akey, J.M. (2015). Complex history of admixture between modern humans and Neandertals. Am. J. Hum. Genet. *96*, 448–453. https://doi.org/10.1016/j.ajhg.2015.01.006.

72. Kim, B.Y., and Lohmueller, K.E. (2015). Selection and reduced population size cannot explain higher amounts of Neandertal ancestry in East Asian than in European human populations. Am. J. Hum. Genet. *96*, 454–461. https://doi.org/10.1016/j.ajhg.2014.12.029.

73. Fu, Q., Hajdinjak, M., Moldovan, O.T., Constantin, S., Mallick, S., Skoglund, P., Patterson, N., Rohland, N., Lazaridis, I., Nickel, B., et al. (2015). An early modern human from Romania with a recent Neanderthal ancestor. Nature *524*, 216–219. https://doi.org/10.1038/nature14558.

74. Nalley, T.K., and Grider-Potter, N. (2017). Functional analyses of the primate upper cervical vertebral column. J. Hum. Evol. *107*, 19–35. https://doi.org/10.1016/j.jhevol.2017.03.010.

75. Boeckx, C. (2017). The language-ready head: Evolutionary considerations. Psychon. Bull. Rev. *24*, 194–199. https://doi.org/10.3758/s13423-016-1087-5.

76. Steele, J., Clegg, M., and Martelli, S. (2013). Comparative morphology of the Hominin and African ape hyoid bone, a possible marker of the evolution of speech. Hum. Biol. *85*, 639–672. https://doi.org/10.3378/027.085.0501.

77. Goodship, J., Cross, I., Scambler, P., and Burn, J. (1995). Monozygotic twins with chromosome 22q11 deletion and discordant phenotype. J. Med. Genet. *32*, 746–748. https://doi.org/10.1136/jmg.32.9.746.

The American Journal of Human Genetics, Volume *111*

Supplemental information

A regulatory variant impacting *TBX1* expression contributes to basicranial morphology in *Homo sapiens*

Noriko Funato, Arja Heliövaara, and Cedric Boeckx

A

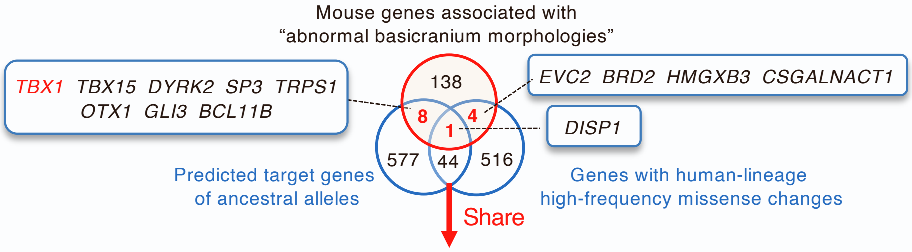

Abnormal basicranium morphologies of gene-engineered mice

Gene	Abnormal basicranium angle	Abnormal foramen magnum morphology	Abnormal basioccipital bone	Abnormal SOS	Abnormal basisphenoid bone	Abnormal ISS	Abnormal presphenoid bone	Short basicranium	References PubMed ID MGI Ref. ID
Evc2	N	N	N	N	+	+	+	+	23026747
Tbx1	+ (this study)	+ (this study)	+	+	+	N	N	+	32442036
Tbx15	N	+	+	N	N	N	N	NA	15652702
Dyrk2	N	N	+	+	+	+	+	+	32758357
Sp3	N	N	N	N	+	N	+	NA	11472836
Trps1	N	N	N	N	+	N	+	NA	31130868
Otx1	N	N	N	N	+	N	+	NA	9077465
Disp1	N	N	N	N	+	N	+	NA	15269168
Gli3	N	N	N	N	+	N	N	NA	11978771
Brd2	N	N	N	N	+	N	N	NA	MGI J:239583
Hmgxb3	N	N	N	N	+	N	N	NA	MGI J:239583
Csgalnact1	N	N	N	+	N	N	N	+	30459452
Bcl11b	N	N	N	N	N	N	N	+	23727454

Posterior ← Skull base → Anterior

B

			Allele Frequencies (1000 Genomes Project)					
SNP	Ref Allele	Alt Allele	Global	African	American	Europe	East Asian	South Asian
rs41300444	A	G	0.0160	0	0.003	0.0139	0.0218	0.043
rs41297812	C	T	0.0176	0.0030	0.003	0.0139	0.0258	0.043
rs72646954	T	C	0.0192	0.0091	0.004	0.0139	0.0258	0.042
rs80179718	A	G	0.0192	0.0091	0.004	0.0139	0.0258	0.042
rs41298798	C	G	0.0166	0	0.003	0.0139	0.0258	0.042
rs8137465	T	C	0.0162	0.0053	0.006	0.0129	0.0169	0.041
rs41300472	T	C	0.0160	0	0.003	0.0149	0.0218	0.042

C

rs41298798 (C/G)

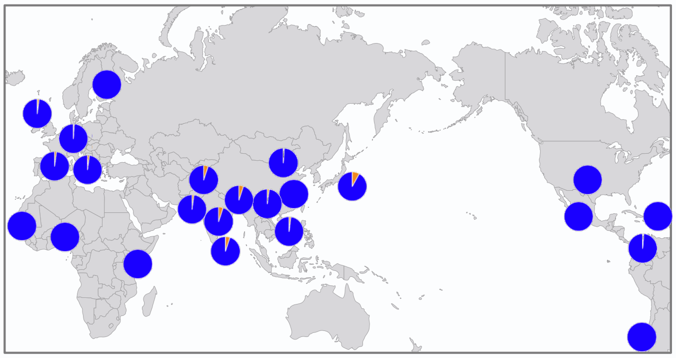

Figure S1. Scheme showing the strategy used to select candidate SNPs.
(A) Strategy for screening candidate genes that induce contemporary-human-specific morphology at the skull base. We used a list of predicted target genes of ancestral alleles,[1] a list of genes with human-lineage high-frequency missense changes,[2] and a list of mouse genes associated with "abnormal basicranium morphologies" obtained from the Mouse Genome Informatics (MGI) database. SOS, spheno-occipital synchondrosis; ISS, intersphenoid synchondrosis; N, not reported; NA, not available.
(B) SNPs at the *TBX1* locus. Ref, reference allele; Alt, alternative allele.
(C) Frequency distribution of rs41298798 in different populations superimposed on a world map. Blue represents the frequency of the C allele. The ancestral G allele (orange) is most common in Japan. Source data are shown in Table S2.

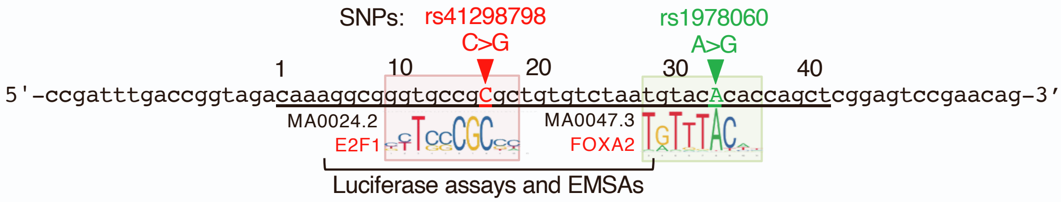

Figure S2. The sequence of a 41-bp deletion at the rs41298798 regulatory region includes rs1978060. Sequence of a 41-bp deletion at rs41298798, with two single-guide RNAs flanking the rs41298798 regulatory region. The variant rs1978060, which confers a significant genetic predisposition to adolescent idiopathic scoliosis in an East Asian population,[3] is located in a 17-bp 3′ region of rs41298798. EMSAs, Electrophoretic mobility shift assays.

rs41298798 rs1978060

1 10 20 30 40

5'-caaaggcgggtgccgCgctgtgtctaatgtacAcaccagct-3'

Matrix ID	Name	Score	Relative score	Start	End	Strand	Predicted sequence
MA0767.1	GCM2	7.807088	0.850760689	3	12	+	aaggcgggtg
MA0745.1	SNAI2	5.510656	0.856435377	5	13	+	ggcgggtgc
MA0024.2	E2F1	5.776952	0.854934316	3	13	+	aaggcgggtgc
MA0522.3	TCF3	9.523167	0.898157425	4	14	-	ggcacccgcct
MA1648.1	TCF12	9.294592	0.888993111	4	14	-	ggcacccgcct
MA0103.3	ZEB1	7.4171805	0.873963857	4	14	-	ggcacccgcct
MA1099.2	HES1	5.41556	0.856389072	5	14	+	ggcgggtgcc
MA0830.2	TCF4	10.570523	0.885564656	3	15	-	cggcacccgcctt
MA1631.1	ASCL1	10.24938	0.880972334	3	15	-	cggcacccgcctt
MA1583.1	ZFP57	10.4456	0.909558656	7	19	+	cgggtgccgCgct
MA0024.2	E2F1	5.5903487	0.852126923	9	19	-	agcGcggcacc
MA1563.1	SOX18	6.839807	0.907395756	15	22	-	cacagcGc
MA1647.1	PRDM4	9.553936	0.88333676	16	26	+	Cgctgtgtcta
MA0442.1	SOX10	7.0969906	0.91952059	18	23	+	ctgtgt
MA0498.2	MEIS1	6.205561	0.912160739	20	26	-	tagacac
MA0033.1	FOXL1	5.5664096	0.857303304	19	26	-	tagacaca
MA0723.2	VAX2	5.5537796	0.892951318	24	31	-	tacattag
MA0032.1	FOXC1	5.460103	0.887481988	24	31	+	ctaatgta
MA0875.1	BARX1	5.9942513	0.885961173	24	31	-	tacattag
MA0876.1	BSX	5.3524723	0.876570725	24	31	-	tacattag
MA1504.1	HOXC4	4.738065	0.876302534	24	31	-	tacattag
MA0132.2	PDX1	5.527857	0.875079817	24	31	-	tacattag
MA0723.2	VAX2	4.216518	0.863018615	24	31	+	ctaatgta
MA1498.2	HOXA7	5.4942107	0.861343583	24	31	-	tacattag
MA0710.1	NOTO	8.469622	0.91750614	23	32	-	gtacattaga
MA0903.1	HOXB3	6.921685	0.895003802	23	32	-	gtacattaga
MA0902.1	HOXB2	6.6082387	0.882637117	23	32	-	gtacattaga
MA0900.1	HOXA2	5.6248317	0.857601958	23	32	-	gtacattaga
MA0035.4	GATA1	7.874824	0.852245545	22	32	+	gtctaatgtac
MA0888.1	EVX2	5.4408298	0.851964332	23	32	-	gtacattaga
MA0849.1	FOXO6	8.234046	0.914004601	29	35	+	gtacAca
MA0157.1	FOXO3	9.379627	0.907205504	28	35	+	tgtacAca
MA0848.1	FOXO4	7.686152	0.90060836	29	35	+	gtacAca
MA0847.1	FOXD2	6.9104347	0.899571311	29	35	+	gtacAca
MA0033.2	FOXL1	5.8368673	0.882262815	29	35	+	gtacAca
MA0042.2	FOXI1	6.2587543	0.866962617	29	35	+	gtacAca
MA0148.2	FOXA1	8.519514	0.86534325	25	35	-	tgTgtacatta
MA0148.1	FOXA1	8.522632	0.864248817	25	35	-	tgTgtacatta
MA0850.1	FOXP3	5.5353866	0.854954653	29	35	+	gtacAca
MA0847.2	FOXD2	10.602455	0.900613175	24	36	+	ctaatgtacAcac
MA0032.1	FOXC1	6.571134	0.949630275	30	37	-	ggtgTgta
MA0047.3	FOXA2	10.523581	0.906065173	27	37	+	atgtacAcacc
MA1103.2	FOXK2	10.31445	0.901509117	27	37	+	atgtacAcacc
MA1683.1	FOXA3	10.009295	0.896010813	27	37	+	atgtacAcacc
MA1103.1	FOXK2	9.9036875	0.892487828	27	37	+	atgtacAcacc
MA0481.3	FOXP1	9.700128	0.892378259	27	37	+	atgtacAcacc
MA0481.2	FOXP1	8.9081	0.860491841	27	38	+	atgtacAcacca
MA0807.1	TBX5	7.183253	0.853436044	31	38	-	tggtgTgt
MA0148.3	FOXA1	10.862134	0.912633721	25	39	-	ctggtgTgtacatta
MA1563.1	SOX18	5.235231	0.86676277	32	39	+	cAcaccag
MA1566.1	TBX3	7.0365567	0.865503127	30	39	-	ctggtgTgta
MA0745.1	SNAI2	7.6557755	0.899224965	33	41	-	agctggtgT
MA1558.1	SNAI1	8.469676	0.898710002	32	41	-	agctggtgTg
MA0830.1	TCF4	6.542236	0.887485653	32	41	+	cAcaccagct
MA0522.2	TCF3	6.4015374	0.877109288	32	41	+	cAcaccagct

rs41298798

rs1978060

Figure S3. The predicted effect of the 41-bp deletion on transcription factor (TF) binding.
A total of 55 putative sites were found with a relative profile score threshold of 85% for all human TF motif sets using the JASPAR 2020 CORE data set.

SNP ID	Variant ID	Gene Symbol	*P*-value	Tissue
rs1978060	chr22_19762002_A_G_b38	*TBX1*	3.00E-18	Prostate
rs1978060	chr22_19762002_A_G_b38	*TBX1*	5.90E-07	Esophagus - Mucosa
rs1978060	chr22_19762002_A_G_b38	*GNB1L*	0.0000076	Brain - Nucleus accumbens (basal ganglia)
rs1978060	chr22_19762002_A_G_b38	*GNB1L*	0.00011	Thyroid
rs1978060	chr22_19762002_A_G_b38	*RTL10 / C22orf29*	0.000016	Brain - Caudate (basal ganglia)
rs1978060	chr22_19762002_A_G_b38	*RTL10 / C22orf29*	1.10E-18	Nerve - Tibial
rs1978060	chr22_19762002_A_G_b38	*AC000089.3*	0.0000036	Nerve - Tibial

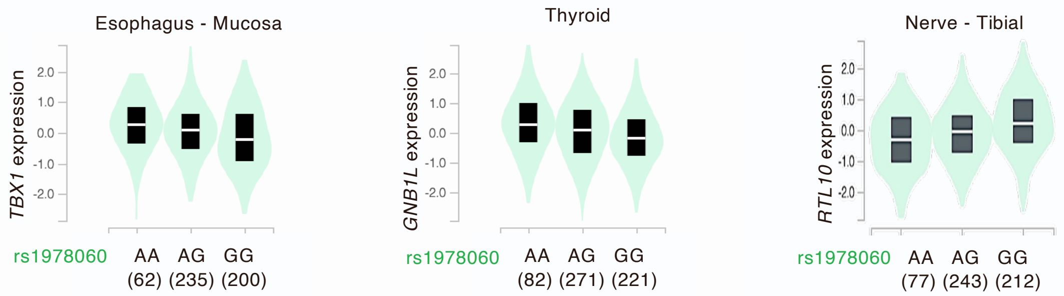

Figure S4. Expression quantitative trait locus (eQTL) analysis of all tissues shows that rs1978060 is associated with the expression levels of *TBX1*, *GNB1L*, *RTL10*, and *AC000089.3*.
GTEx was accessed on 29 June 2022.

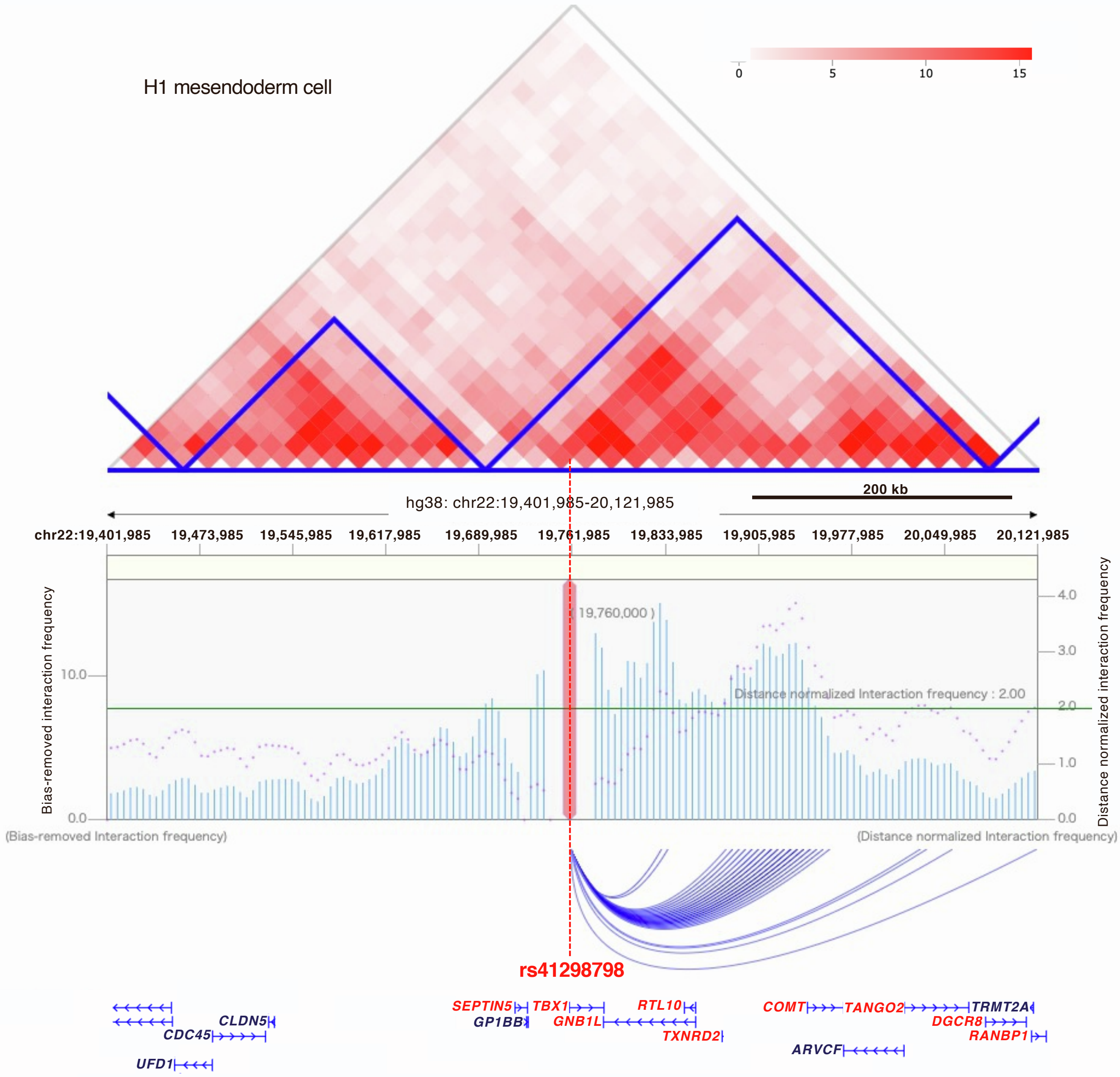

Figure S5. Contact between rs41298798 and genes at 22q11.21 based on the three-dimensional chromatin structure. Snapshot of the 3D Interaction Viewer (3DIV) with the hg38 genome assembly showing the genomic context of chromosome 22q11.21.

(Top) 20 kb resolution Hi-C data in H1 mesendoderm cells.

(Bottom) Coding RNAs. Strong interactions were observed between the region containing rs41298798 and promoters in the same topologically associating domain (blue triangle box). Genes shown in red have been analysed in Figure 2.

A

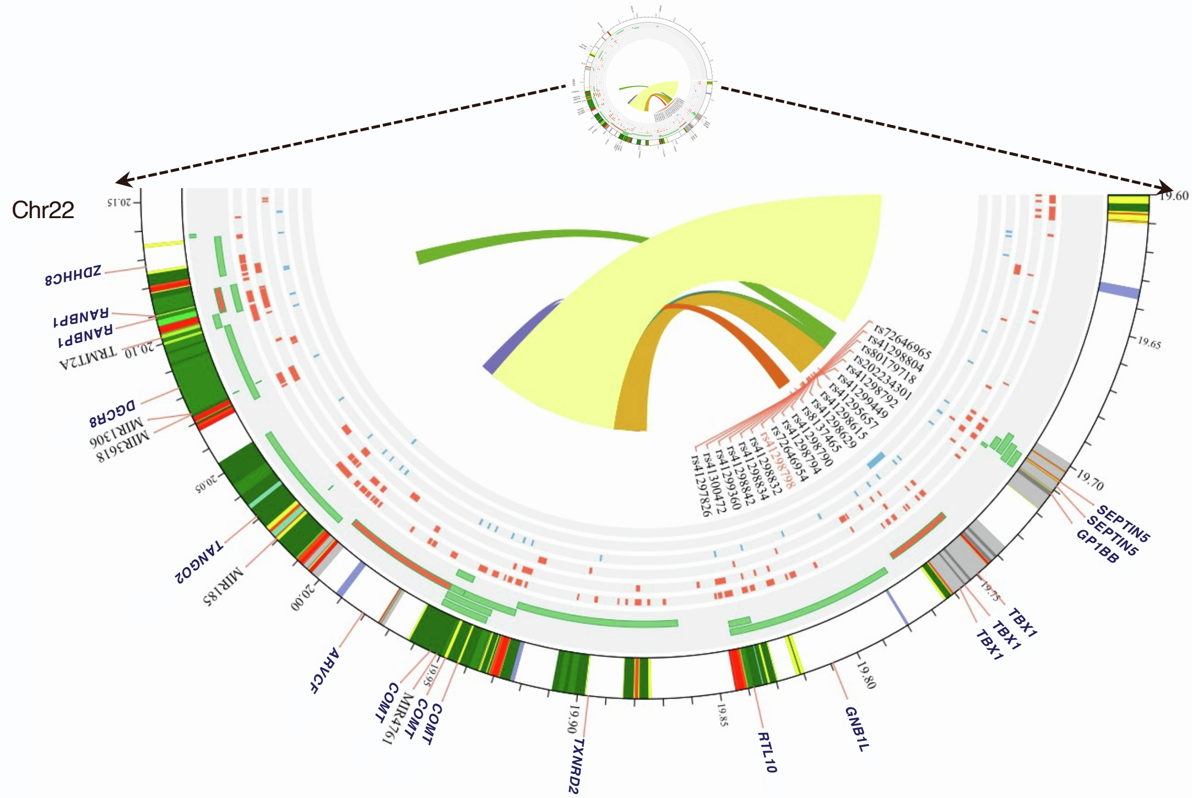

B

Gene	Loop type	Cell line	Tissue
SEPTIN5	Within loop	LNCAP	
SEPT5-GP1BB	Within loop	LNCAP	
GP1BB	Within loop	LNCAP GM12878 HepG2	Blood, Liver
TBX1	Within loop	SKMEL5 LNCAP THP1 HAP1 HepG2 KBM7 NHEK PANC1 GM12878 HCT116 IMR90 HMEC Caki2 H1-NPC HUVEC	Liver, Skin, Blood, Lung, Breast, Spleen, Pancreas, Blood vessel
GNB1L	Within loop	SKMEL5 THP1 HAP1 HepG2 T47D RPMI7951 NHEK PANC1 GM12878 HCT116 IMR90 HMEC Caki2 H1-NPC KBM7 A549 HUVEC	Liver, Psoas, Skin, Blood, Aorta, Spleen, Pancreas, Lung, Breast, Epithelium, Blood vessel
RTL10	Within loop	SKMEL5 THP1 HAP1 HepG2 T47D RPMI7951 NHEK PANC1 GM12878 LNCAP HCT116 IMR90 HMEC Caki2 H1-NPC KBM7 A549 HUVEC	Psoas, Lung, Liver, Skin, Aorta, Spleen, Pancreas, Blood, Breast, Epithelium, Blood vessel
TXNRD2	Within loop	LNCAP	
COMT	Within loop	LNCAP	

C

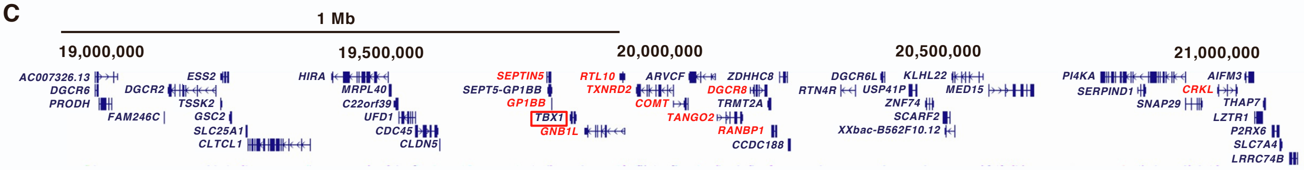

Figure S6. Long-range chromatin looping formed between the enhancer region containing the candidate causal SNP rs41298798.

(A) A circular plot of chromosome interactions related to rs41298798. In the circular plot, from outer to inner, the circle represents chromatin states, annotated genes, histone modification set (red; H3K4me1, H3K4me3, H3K27ac), TF set (blue; CTCF, CEBPB, CEBPD), current SNP (red) and associated SNPs, and three-dimensional (3D) chromatin interactions.[4]

(B) Genes that interact with rs41298798 through 3D chromatin loops in different cell types and tissues. Data were taken from 3DSNP v2.1.

(C) Genes at the 22q11.21 locus. Genes shown in red have been analysed in Figure 2.

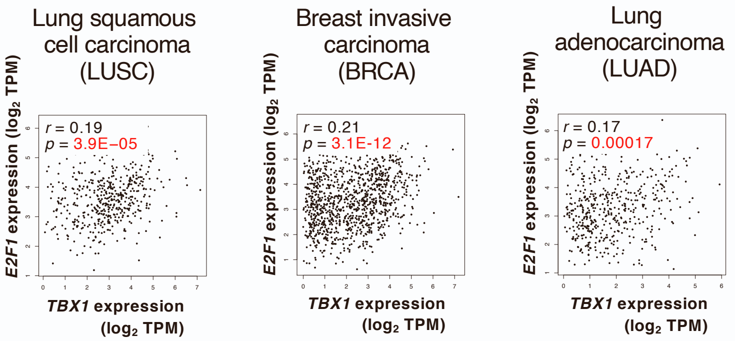

Figure S7. *E2F1* expression levels are positively correlated with *TBX1* expression levels.
Pearson correlations (r) between *E2F1* and *TBX1* expression levels in The Cancer Genome Atlas database.

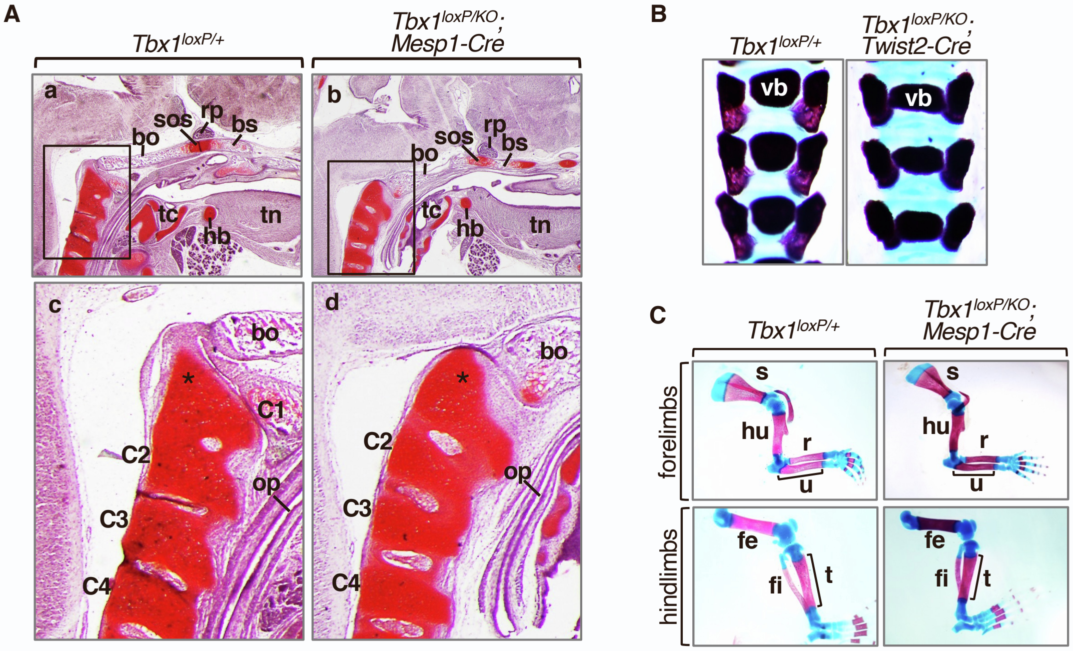

Figure S8. ***Tbx1**$^{loxP/KO}$**;Mesp1-Cre*** **and** ***Tbx1**$^{loxP/KO}$**;Twist2-Cre*** **mice recapitulate the phenotypes of** ***Tbx1*****-knockout mice.**

(A) Sagittal sections of E16.5 control (a,c) and *Tbx1$^{loxP/KO}$;Mesp1-Cre* (b,d) embryos were stained with safranin O/hematoxylin/Fast Green and observed at low (a,b) and high (c,d) magnification. Asterisk, the odontoid process of C2. bo, the primordium of the basioccipital bone; bs, the primordium of the basisphenoid bone; sos, spheno-occipital synchondrosis; rp, Rathke's pouch; hb, hyoid bone; tc, thyroid cartilage; tn, tongue; op, oropharynx.

(B) Ventral view of lumbar vertebrae (L4–L6) of control and *Tbx1$^{loxP/KO}$;Twist2-Cre* neonates stained for bone and cartilage. vb, vertebral body.

(C) Bone staining of forelimbs and hindlimbs of control and *Tbx1$^{loxP/KO}$;Mesp1-Cre* neonates. Brackets indicate the ossified shaft of the ulna (u) and tibia (t). s, scapula; hu, humerus; r, radius; fe, femur; fi, fibula.

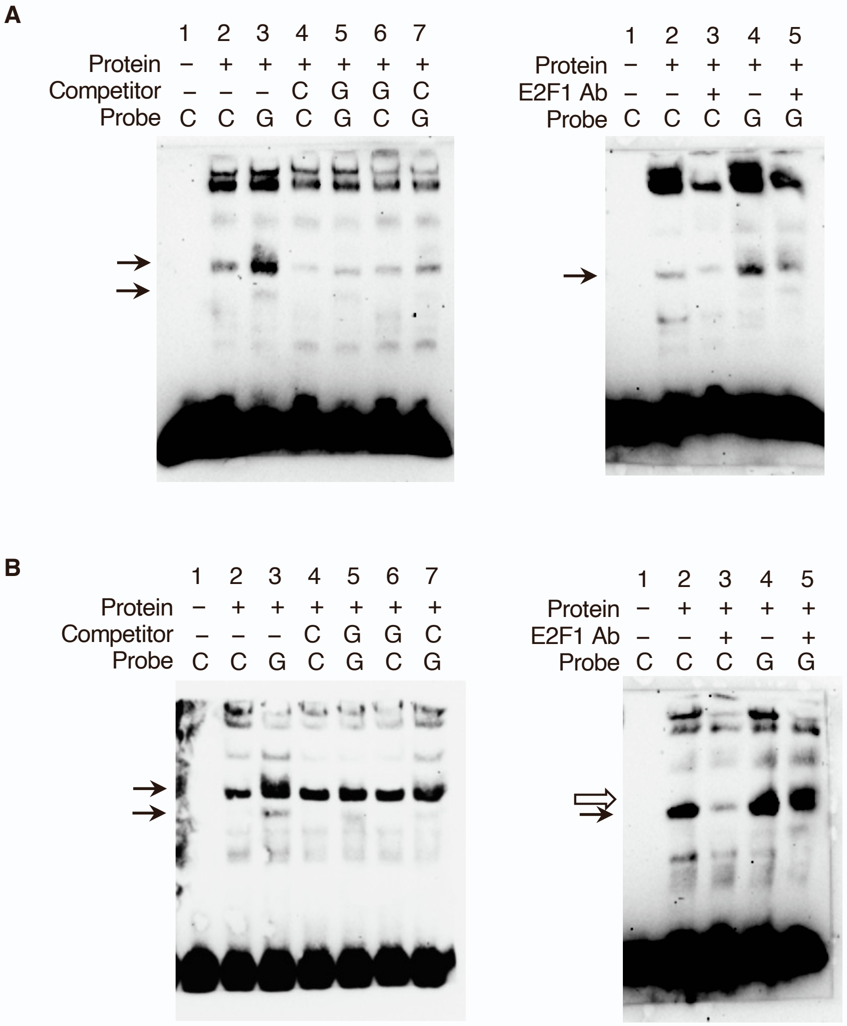

Figure S9. Full-length images of representative electrophoretic mobility shift assays related to Figure 3D. (A,B) Electrophoretic mobility shift assays (EMSAs) with biotin-labelled probes containing rs41298798-[C] or rs41298798-[G] alleles in E2F1-transfected COS1 (A) and HeLa cells (B). Black arrows, allele-specific bands that interact with nuclear proteins; white arrow, a super-shifted E2F1 complex.

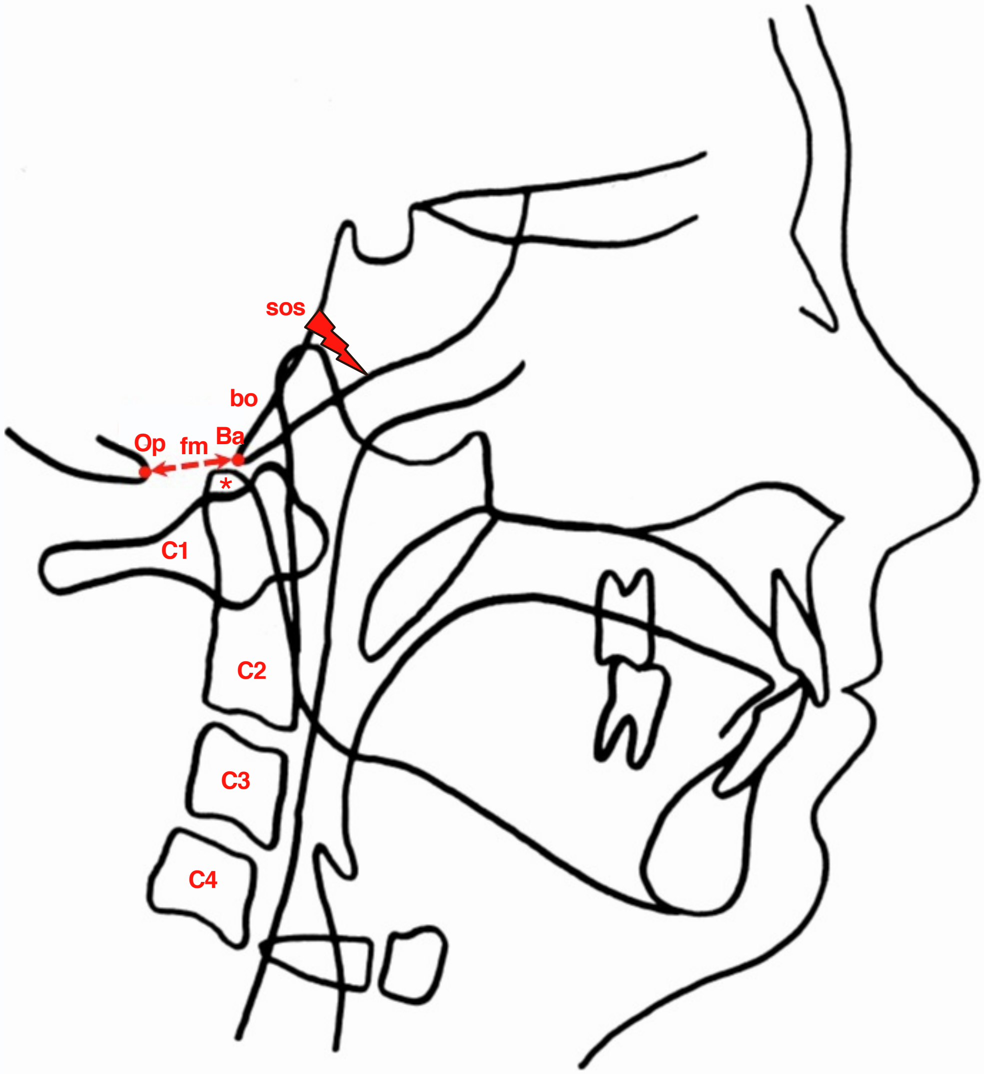

Figure S10. Cephalometric landmarks used in this study.
Ba: Basion, the lowest point on the anterior border of the foramen magnum (fm); Op: Opisthion, the posterior border of the foramen magnum; bo, basioccipital bone; sos, spheno-occipital synchondrosis; asterisk, C2 odontoid process.

Supplemental Methods

A list of mouse genes associated with abnormal basicranium morphologies.

Basicranium morphology	Gene
Abnormal basicranium angle	*Bmp7 Smg1*
Abnormal foramen magnum morphology	*Ank Fgfr3 Fgfrl1 Foxc1 Idua Ltbp3 Pth1r Tbx15 Tbx1*
Abnormal basioccipital bone morphology	*Ank Arl6 Baiap2 Bmp7 Cdx1 Chrd Ctnnb1 Dlx5 Dyrk2 Evc Fgfr2 Fgfrl1 Foxc2 Foxd3 Foxn3 Gli2 Hhat Hoxa1 Hoxa2 Hoxa3 Hoxd4 Ltbp1 Ltbp3 Memo1 Nedd4 Nkx3-2 Pcgf2 Pgap1 Psen1 Rara Rarb Rarg Rpgrip1l Rr45 Sh3pxd2a Ski Tbx1 Tbx15 Tmem107 Wnt9a*
Abnormal basisphenoid bone morphology	*Adamts3 Alx1 Alx4 Ankrd11 Apaf1 Arl6 Baiap2 Bmp7 Bmper Boc Brd2 Cdon Chrd Coro1c Crkl Ctnnb1 Dhx35 Dicer1 Disp1 Dlx2 Dlx5 Dlx6 Dyrk2 Msx1 Snrpb Ednra Evc Evc2 Eya1 Fgfr1 Fgfr2 Fgfrl1 Foxc1 Foxc2 Foxn3 Gas1 Gas2l2 Shh Gli2 Gli3 Gm5544 Kmt2d Memo1 Srsf3 Hand1 Hmgxb3 Hoxa2 Kif3a Ltbp1 Ltbp3 Memo1 Men1 Mn1 Morc2a Nkx3-2 Nosip Nsun2 Otx1 Otx2 Pdgfra Pkdcc Prtg Ptch1 Ptprf Ptprs Rara Rarg Rpgrip1l Rr45 Rr115 Shh Runx2 Sh3pxd2a Ski Smg9 Smo Sox9 Sp3 Ssr2 Stk11 Sulf1 Sulf2 Tbx1 Tcf7l2 Tmem107 Trps1 Wnt1 Wnt3a Zeb1*
Abnormal presphenoid bone morphology	*Alx1 Alx4 Apaf1 Arid5b Arl6 Bmp2 Chrd Chuk Csrnp1 Dicer1 Dlx5 Dlx6 Dyrk2 Fgfr2 Fgfr3 Foxc2 Foxf2 Gli2 Gsc Kmt2d Memo1 Irf6 Lmo4 Mks1 Mn1 Otx1 Otx2 Phc1 Phc2 Pkd1 Ptch1 Rara Rarg Rr133 Schip1 Sgpl1 Ski Smo Sox9 Sp3 Stk11 Tiparp Tmem107 Trps1 Vax1 Wnt1 Wnt3a Zfp640 Zfp950*
Abnormal spheno-occipital synchondrosis (SOS)	*Arl6 Csgalnact1 Fgfr2 Pkd1 Tbx1 Chrd Ctnnb1 Fgfrl1 Ihh Mef2c Pth1r Six1 Six4 Chrd Por Nppc Twist1 Lef1 Pthlh Runx2 Six2 Kif3a Hdac4 Map2k1 Id2 Ift88 Alpl Ltbp3 Fgfr3*
Abnormal intersphenoid synchondrosis (ISS)	*Arl6 Evc Evc2 Fgfr2 Pkd1 Pkd2 Lef1 Ctnnb1 Pthlh Runx2 Six2 Kif3a Hdac4 Map2k1 Id2 Ift88 Alpl Ltbp3 Fgfr3*
Short basicranium	*Acan Arl6 B3glct Bcl11b Bmp7 Fgfr2 Fgfr3 Foxc1 Hapln1 Ltbp3 Mbtps1 Rr23 Rr25 Src*
Premature cranial synchondrosis closure	*Fgfr3 Pfas Pkd1*

A list of mouse genes associated with “abnormal basicranium morphologies” was obtained from the Mouse Genome Informatics (MGI) database[5] and the published review.[6] See Table S5 for full information on the MGI database.

Sequences for single-guide RNA cloning and validation.

sgRNA	Sequence	PAM
5′ Δ41 Guide 1	5′-CCGATTTGACCGGTAGACAA-3'	5′-AGG-3'
3′ Δ41 Guide 2	5′-TGGCTGTTCGGACTCCGAGC-3'	5′-TGG-3'
Precise editing	5′-TGGCTGTTCGGACTCCGAGC-3'	5′-TGG-3'
**Genotyping primers**	**Sequence**	
Left primer	5′-TAAAGACCAAGAGACAAGGGGA-3'	
Right primer	5′-AAACAGAAACCACGAGAAGGCT-3'	
**ssODN**	**Sequence**	
ssODN-C	5′-caagttttgcagatgcacccgatttgaccggtagacaaaggcgggtgccgCgctgtgtctaatgtacacaccagctcg gagtccgaacagccaaggggagc-3'	
ssODN-G	5′-caagttttgcagatgcacccgatttgaccggtagacaaaggcgggtgccgGgctgtgtctaatgtacacaccagctcg gagtccgaacagccaaggggagc-3'	

Sequences of primers used for quantitative polymerase chain reaction.

Gene	Sense Sequence	Antisense Sequence
TBX1	5'-TAGCGAGAAATATGCCGAGGA-3'	5'-CGTGATCCGATGGTTCTGGT-3'
RANBP1	5'-AATACAGACGAGTCCAACCATGA-3'	5'-GAACAGTTTTGCCCGCATTTTA-3'
GNB1L	5'-CGGCTATGAGGATGGATCG-3'	5'-CTGGGAGTCAAAGTCAAGGTC-3'
TANGO2	5'-CTGGCAGCACTCACCAACTAC-3'	5'-GTCAGTGGTCAGAAAGTGGGT-3'
COMT	5'-GAAGGGGACAGTGCTACTGG-3'	5'-CAGGAACGATTGGTAGTGTGTG-3'
ARVCF	5'-CTATTGTCACATCCGAAGATGGC-3'	5'-CGTACTGTCCGAGTGGTCAC-3'
TXNRD2	5'-CTAGCCCCGACACTCAGAAGA-3'	5'-GGCCATGATCGCTATGGGT-3'
RTL10	5'-GTTTTGCTGAGTACCATGCTGT-3'	5'-GCCAGGCCCTCTAAGAACC-3'
SEPTIN5	5'-CGCATCAGCCAGACGGTAG-3'	5'-CCGCTCTCATCACGGAAGT-3'
DGCR8	5'-GCAGAGGTAATGGACGTTGG-3'	5'-AGAGAAGCTCCGTAGAAGTTGAA-3'
CRKL	5'-CTGTCGGTGTCCGAGAACTC-3'	5'-TGGTCCCCGATCTTAAAACGG-3'
GAPDH	5'-GACAGTCAGCCGCATCTTCT-3'	5'-GCGCCCAATACGACCAAATC-3'

Supplemental References

1. Peyregne, S., Boyle, M.J., Dannemann, M., and Prufer, K. (2017). Detecting ancient positive selection in humans using extended lineage sorting. Genome Res. *27*, 1563–1572. 10.1101/gr.219493.116.
2. Kuhlwilm, M., and Boeckx, C. (2019). A catalog of single nucleotide changes distinguishing modern humans from archaic hominins. Sci. Rep. *9*, 8463. 10.1038/s41598-019-44877-x.
3. Kou, I., Otomo, N., Takeda, K., Momozawa, Y., Lu, H.-F., Kubo, M., Kamatani, Y., Ogura, Y., Takahashi, Y., Nakajima, M., et al. (2019). Genome-wide association study identifies 14 previously unreported susceptibility loci for adolescent idiopathic scoliosis in Japanese. Nat. Commun. *10*, 3685. 10.1038/s41467-019-11596-w.
4. Yang, D., Jang, I., Choi, J., Kim, M.S., Lee, A.J., Kim, H., Eom, J., Kim, D., Jung, I., and Lee, B. (2018). 3DIV: A 3D-genome Interaction Viewer and database. Nucleic Acids Res. *46*, D52–D57. 10.1093/nar/gkx1017.
5. Blake, J.A., Baldarelli, R., Kadin, J.A., Richardson, J.E., Smith, C.L., Bult, C.J., and Mouse Genome Database Group (2021). Mouse Genome Database (MGD): Knowledgebase for mouse-human comparative biology. Nucleic Acids Res. *49*, D981–D987. 10.1093/nar/gkaa1083.
6. Funato, N. (2020). New insights into cranial synchondrosis development: A mini review. Front. Cell Dev. Biol. *8*, 706. 10.3389/fcell.2020.00706.
